# The Formation of Inherently Chiral Calix[4]quinolines by Doebner–Miller Reaction of Aldehydes and Aminocalixarenes

**DOI:** 10.3390/molecules27238545

**Published:** 2022-12-04

**Authors:** Martin Tlustý, Václav Eigner, Hana Dvořáková, Pavel Lhoták

**Affiliations:** 1 Department of Organic Chemistry, University of Chemistry and Technology, Prague (UCTP), Technická 5, 166 28 Prague, Czech Republic; 2 Department of Solid State Chemistry, UCTP, Technická 5, 166 28 Prague, Czech Republic; 3 Laboratory of NMR Spectroscopy, UCTP, Technická 5, 166 28 Prague, Czech Republic

**Keywords:** calixarene, inherent chirality, complexation, mercuration, *meta*-substitution, quinoline formation, chiral recognition

## Abstract

The formation of inherently chiral calix[4]arenes by the intramolecular cyclization approach suffers from a limited number of suitable substrates for these reactions. Here, we report an easy way to prepare one class of such compounds: calixquinolines, which can be obtained by the reaction of aldehydes with easily accessible aminocalix[4]arenes in acidic conditions (Doebner–Miller reaction). The synthetic procedure represents a very straightforward approach to the inherently chiral macrocyclic systems. The complexation studies revealed the ability of these compounds to complex quaternary ammonium salts with different stoichiometries depending on the guest molecules. At the same time, the ability of enantioselective complexation of chiral *N*-methylammonium salts was demonstrated.

## 1. Introduction

**Figure 1:**
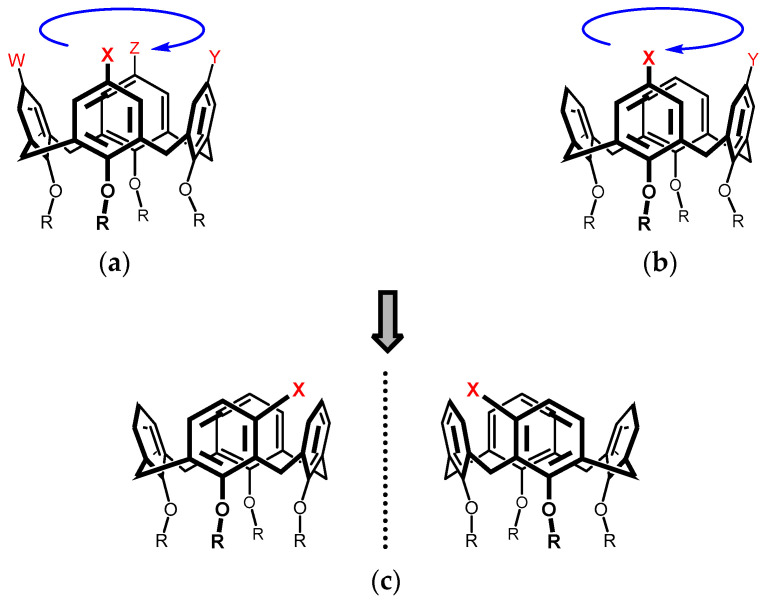
Examples of inherently chiral calix[4]arenes: (**a**) WXYZ pattern; (**b**) XY pattern; (**c**) *meta* substitution.

Calixarenes [1,2,3,4] are macrocyclic compounds which are widely used in supramolecular chemistry. The reason for their popularity can be found in the combination of several factors: easy multi-gram preparation, variable size and tuneable shape of the cavity, simple derivatization, etc. Depending on the substitution, calixarenes exhibit good complexation properties towards cations, anions or neutral compounds [5,6,7,8,9]. However, unlike some other macrocycles (e.g., cyclodextrins, pillararenes), calixarenes themselves are achiral molecules, which makes them useless as chiral receptors without further derivatization. One possible solution to chirality issues is to convert calixarenes into so-called inherently chiral systems [10,11,12], where the combination of nonplanar molecules with suitable substitution patterns can lead to chirality without the introduction of stereogenic units. For example, calix[4]arenes in the *cone* conformation with WXYZ (Figure 1a) or XY (Figure 1b) substitution patterns (upper rim or lower rim) exhibit such chirality because the presence of different substituents gives the system a particular sense of rotation (Figure 1a). Similarly, the *meta* substitution [13] of calix[4]arenes yields chiral compounds (Figure 1c) and, as such, represents the most straightforward approach to such systems. However, an obvious drawback of this approach is the lack of corresponding derivatization techniques enabling selective substitution of the *meta* position. In fact, the electrophilic aromatic mercuration is so far the only procedure known to provide *meta* substituted products from an unsubstituted calix[4]arenes [14].

**Figure 2:**
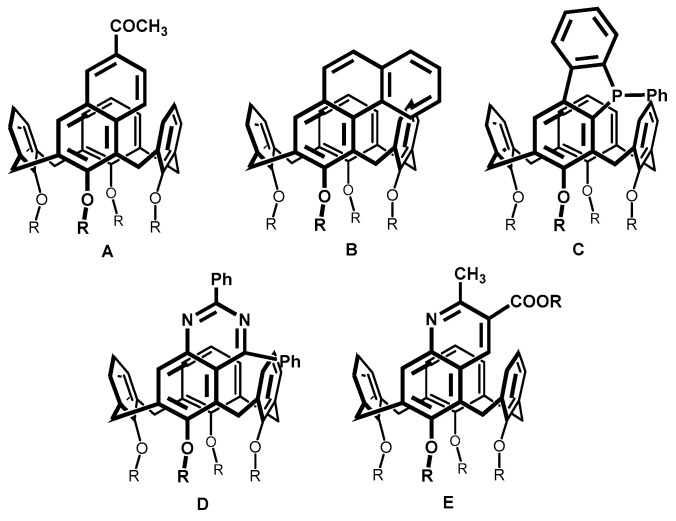
Examples of calix[4]arenes with fused rings.

Intramolecular cyclization represents a different way for the synthesis of inherently chiral *meta* substituted calixarenes. This approach consists of two steps: (i) the introduction of a suitable functional group to the *para* position of calixarene; and (ii) intramolecular cyclization, leading to calixarenes with fused rings. As an example, we can mention the preparation of the naphthalene moiety (A) starting from an aldehyde [15], phenanthrene moiety (B) by the photocyclization of stilbene [16], the formation of calixarene-fused phosphols [17] (C) and the preparation of calixquinazolines (D) (Figure 2) [18]. However, most of these approaches suffer from a complicated synthesis of starting compounds or a low-yielding cyclization step. Miao et al. [19] also synthesized calixquinolines (E); however, this approach required the utilization of bifunctional reagents (crotonaldehyde or ethyl acetoacetate).

In this paper, we report on the synthesis of calixquinolines from easily accessible aminocalix[4]arenes derivatives (both *meta*- and *para*-) in the *cone* conformation by the tandem reaction with simple aldehydes (acetaldehyde, bromoacetaldehyde). Although the reaction conditions were initially designed for the Pictet–Spengler condensation [20,21] of *meta*-aminocalixarene with aldehydes, the unexpected formation of calixquinolines (Doebner–Miller reaction) turned out to be general and worked with *para*-aminocalixarenes as well. This synthetic procedure represents a very straightforward approach to calixquinoline derivatives representing the inherently chiral macrocyclic systems.

## 2. Results and Discussion

**Scheme 1:**
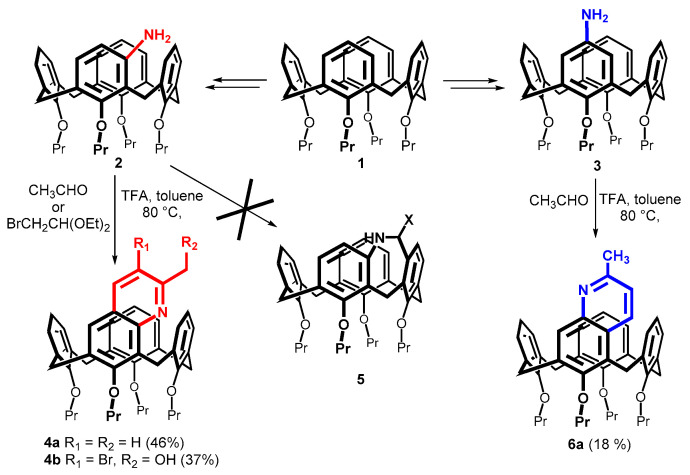
Synthesis of calix[4]quinolines from *meta*- and *para*-aminosubstituted calix[4]arenes.

Our original intention was to prepare amine-bridged macrocycles **5** starting from the *meta*-aminocalix[4]arenes (Scheme 1). These compounds would represent a reduced form of our recently reported imine-bridged calixarenes [22,23]. Using the known procedure, 4-aminocalix[4]arenes **2** was prepared in three steps from the starting calixarene **1** (Scheme 1). Thus, the initial mercuration of **1** with one equivalent of Hg(TFA)_2_ gave the corresponding *meta* HgCl derivative in 65% yield [14]. Subsequent reaction with isoamyl nitrite/HCl gave nitroso compound (91%) [24], which was finally reduced by N_2_H_4_/Ni(R) to the desired amine **2** (89%) [22].

Surprisingly, the Pictet–Spengler reaction of calixarene **2** with aliphatic aldehydes did not provide the expected amine-bridged compounds **5**. Conversely, the reaction of amine **2** with acetaldehyde in the presence of TFA in toluene at 80 °C gave the quinoline derivative **4a** in 46% yield (Scheme 1). Similarly, the same reaction conditions and bromoacetaldehyde diethyl acetal gave compound **4b** in 37% yield. The unexpected hydroxy group was probably introduced into the molecule by the substitution of the bromine atom during the workup. The formation of these compounds can be explained by a Doebner–Miller reaction [25,26] consisting of the initial aldol reaction, followed by the conjugate addition and the final ring closure. Indeed, benzaldehyde, which does not work as a substrate in aldol reactions, provided a complex mixture of products. The same holds for the ketones acetone and acetophenone. These results indicated that only aliphatic aldehydes are suitable for this type of reaction.

To test the general applicability within the calixarene series, the *para*-amino-substituted derivative **3** was prepared by the nitration of starting **1** with 100% HNO_3_ in the presence of glacial acetic acid in dichloromethane [27] and the subsequent reduction of nitro intermediate with SnCl_2_ [28]. The reaction with acetaldehyde carried out under identical conditions as for **2** (TFA in toluene at 80 °C) provided quinoline derivative **6a** in 18% yield (Scheme 1).

The structure of compound **4a** was confirmed by the combination of NMR and HRMS techniques. The ^1^H NMR spectrum (400 MHz, CDCl_3_) showed the presence of two sets of four doublets for the methylene bridge protons at 5.03, 4.66, 4.52, 4.51 and 4.35, 3.37 and 3.19 (2×) with typical geminal coupling constants (~13.5 Hz) consistent with the *C_1_*-symmetrical calix[4]arenes. The spectrum also contained a singlet at 2.76 ppm, revealing the presence of a methyl group. Moreover, the presence of a significantly downfield-shifted doublet at 8.00 ppm suggested the presence of a strong electron-withdrawing group (nitrogen) within the aromatic structure. The HRMS ESI^+^ analysis showed signals at *m/z* = 658.3892 and 680.3705 corresponding to [M+H]^+^ (658.3891) and [M+Na]^+^ (680.3710) predicted for **4a**.

**Scheme 2:**
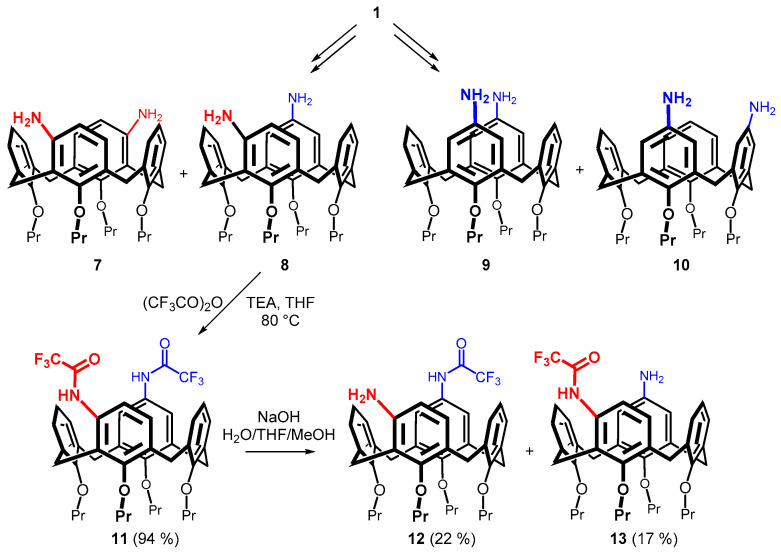
Synthesis of *meta*- and *para*-aminosubstituted calix[4]areness.

As the above synthetic protocol represents a very straightforward approach to inherently chiral calixarene derivatives, we decided to prepare a library of diaminocalixarenes, which are synthetically available (Scheme 2).

The starting tetrapropoxycalix[4]arenes **1** was transformed into diamines **7** and **8** using the procedure described by our group. Briefly, a reaction with two equivalents of Hg(TFA)_2_ in chloroform provided a mixture of two distally dimercurated calixarenes: *meta,meta* and *meta,para* isomers [29]. The mixture was reacted with isoamyl nitrite in the presence of HCl and AcOH in chloroform to yield the corresponding nitroso derivatives, which are easily separated by column chromatography on silica gel. The resulting amines **7** and **8** were obtained by reduction with hydrazine in the presence of Raney-nickel in refluxing ethanol [23]. To synthesize the *para*-diamino derivatives **9** and **10**, calix[4]arene **1** was nitrated with 100% HNO_3_ in glacial acetic acid and dichloromethane as a solvent [27]. The corresponding distal and proximal dinitro derivatives were separated by column chromatography and finally reduced by SnCl_2_ in refluxing toluene [28].

Compound **8** represents a rather unusual structural motif as it contains both *para-* and *meta*-amino groups within the molecule. In order to study possible differences in the reactivity of the two regioisomeric groups, the substance was reacted with an excess of (CF_3_CO)_2_O in the presence of TEA in THF to form diamide **11** in 94% yield (Scheme 2). A careful hydrolysis of this compound finally gave monoamines **12** (*meta*) and **13** (*para*) in 22 and 17% yield, respectively, after a column chromatography on silica gel [23].

**Scheme 3:**
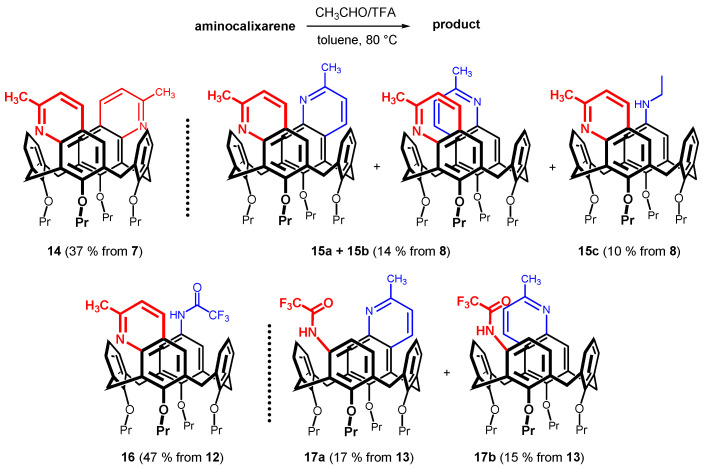
Synthesis of calix[4]quinolines (the number of starting aminocalixarene in brackets); reaction conditions: acetaldehyde, TFA, toluene, 80 °C.

Not all of the amino derivatives shown in Scheme 2 proved useful for the preparation of calixquinolines. Thus, the reaction of acetaldehyde with calixarene **7** (*meta, meta* substitution) gave quinoline **14** in 37% yield (Scheme 3). Unlike the previous example, the cyclization of amine **8** provided a mixture of the two regioisomers/diastereoisomers **15a** and **15b** in low yield (14%). Unfortunately, the mixture was not separable using conventional separation techniques (column chromatography, preparative TLC, flash chromatography). On the other hand, the unexpected by-product **15c** possessing ethylamino group in the *para*-position was also isolated in 10% yield.

**Figure 3:**
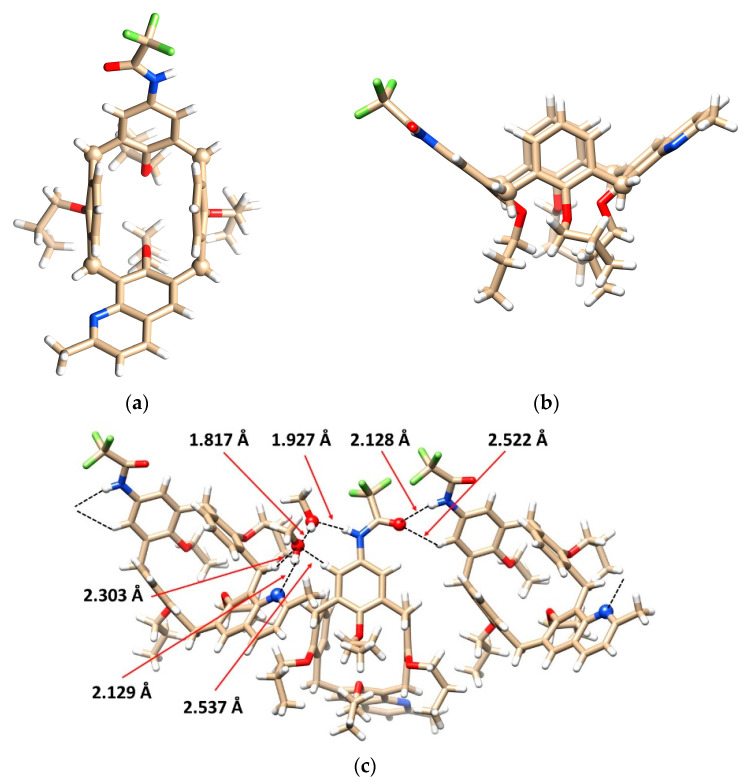
X-ray structure of **16**: (**a**) top view showing *pinched cone* conformation (bridging C atoms shown as balls for better clarity); (**b**) side view of the same; (**c**) the binding motif in crystal packing of enantiomers; methanol solvate was removed for better clarity.

Although cyclization of diamines **9** and **10** should provide only 2 or 3 regioisomers, respectively, in both cases the reaction with acetaldehyde turned out to be much more complicated, and we were unable to isolate any expected quinoline product from complex reaction mixtures. In contrast, *meta*-amine **12** smoothly provided quinoline **16** in 47% yield (Scheme 3). The cyclization of its *para*- congener **13** resulted in the formation of two regioisomers **17a** and **17b**, which were isolated by column chromatography on silica gel in 22% and 17% yields, respectively. The unambiguous proof of the structure of **16** was obtained from single crystal X-ray analysis. The monocrystals of **16** were obtained from dichloromethane/MeOH mixture as a methanol solvate (1:1) in the triclinic system, *P-1* space group. As shown in Figure 3a,b the macrocycle adopts the *pinched cone* conformation, which is common for solid-state structures of the *cone* isomers. Defining the main plane of the calixarene by the four bridging carbon atoms (C2, C8, C14, C20), the aromatic moiety bearing trifluoroacetamide is tilted out of the cavity with the interplanar angle *Φ* = 131.40°. The opposite aromatic subunit with quinoline moiety exhibits similar geometrical parameters (*Φ* = 127.36°). The remaining two phenolic subunits cross the main plane at almost right angles (*Φ* = 79.48° and 84.75°) and are directed slightly into the cavity. This arrangement with large substituents on the subunits facing out of the cavity is apparently the result of steric hindrance minimization (Figure 3b).

Interesting supramolecular interactions were found within the crystal packing of **16**. As shown in Figure 3c, the opposite enantiomers of **16** are held together via hydrogen bond interactions between the carbonyl oxygen and NH groups from amidic functions (NH···O = C distance = 2.128 Å). This bonding is further strengthened by the concomitant HB interaction between the carbonyl and *ortho* hydrogen of aromatic subunit (CH···O = C = 2.522 Å). Interestingly, methanol is included in the form of dimer with OH···O distance of 1.817 Å, indicating a strong HB between methanol molecules (Figure 3c). This dimer is held at both ends by HBs from quinoline nitrogen (N···HO = 2.129 Å) and from amidic NH moiety (NH···O = 1.927 Å).

**Figure 4:**
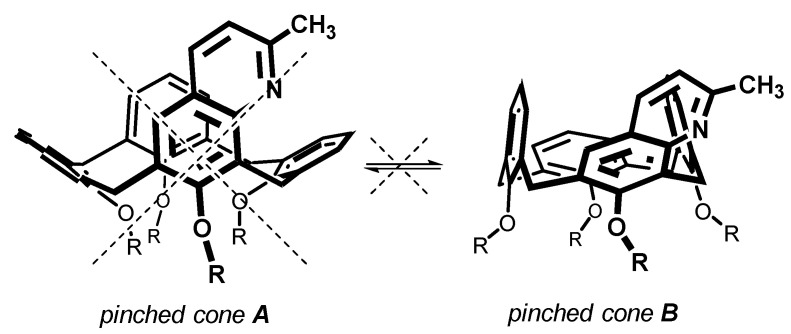
*Pinched cone—pinched cone* interconversion in calix[4]arenes (shown for compound **4a**).

Common lower rim peralkylated calix[4]arenes immobilized in the *cone* conformation are known to exhibit so called *pinched cone—pinched cone* interconversion in solution, where two border conformations mutually equilibrate (Figure 4). The above X-ray analysis revealed that the introduction of heterocyclic moiety into the upper rim of calix[4]arenes leads to a single *pinched cone* conformer in the solid state with substituted aromatic subunits pointing out of the cavity. This means that only one of the two theoretically possible *pinched cone* conformations is preferred, obviously as a consequence of the steric hindrance minimization within the upper rim.

To show the general behaviour of our products in solution, compounds **4a** and **6a** representing the *meta*- and *para*-amino substituted calixarene systems were selected for dynamic ^1^H NMR study. As shown in ESI (Figures S45–S50), both compounds **4a** and **6a** did not show any changes within the whole temperature range studied (298-173 K, CD_2_Cl_2_, 500 MHz). This is strong evidence that both compounds also exist in solution in only one thermodynamically preferred *pinched cone B* conformation as a direct consequence of the attachment of the heterocyclic moiety.

As all quinoline derivatives described above represent the inherently chiral systems, we have carried out a study of their possible resolution on a chiral column. On an analytical scale, the separation of racemic **4a** into enantiomers was feasible with Chiralpak IA (250 × 4.6 ID, 5 µm) column, using heptane/ethyl acetate (95/5, *v*/*v*) as a mobile phase. However, these conditions turned out to be unsuitable for the separation in a preparative scale. On the other hand, the preparative resolution of compound **6a** was successfully carried out using a polysaccharide column ChiralArt Amylose-SA (250 × 20 mm ID, 5 μm) with cyclohexane/DCM: 92/8 *v/v* as a mobile phase. The resulting individual enantiomers **6a_1** and **6a_2** were used for titration study.

**Figure 5:**
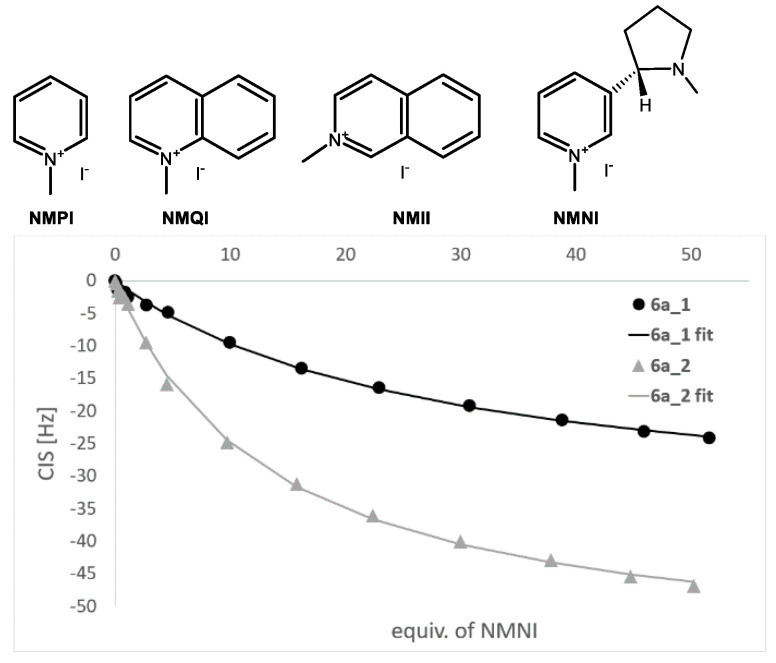
^1^H NMR titration curve of **6a_1** and **6a_2** with NMNI (C_2_D_2_Cl_4_, 400 MHz, 298 K), hydrogen in position 3- of calixarene quinoline moiety observed.

Calixquinolines represent systems with enlarged aromatic cavities potentially capable of interacting with molecules bearing an acidic CH_3_ group (C-H···π interactions). The ^1^H NMR titration experiments revealed the possible use of newly prepared compounds as receptors for ammonium salt complexation (Figure 5). The complexation constant was determined by analyzing the complexation-induced shifts (CIS) of host signals (hydrogen in position 3- of quinolinium moiety) using a nonlinear curve-fitting procedure (program BindFit) [30]. The titration (C_2_D_2_Cl_4_) of **4a** with *N*-methylquinolinium iodide (NMQI) and *N*-methylisoquinolinium iodide (NMII) revealed the formation of 1:1 complexes with complexation constants 12.2 and 16.6 M^−1^, respectively (Standard deviations for NMR titrations are generally estimated to be around 10%). Surprisingly, the titration by a structurally similar *N*-methylpyridinium iodide (NMPI) showed the formation of complexes with 2:1 stoichiometry and complexation constants *K_11_* = 13.0 M^−1^ and *K_21_* = 2798 M^−1^.

Based on these results, we attempted the enantioselective recognition of (*S*)-*N*-methylnicotinium iodide (NMNI) with separated enantiomers **6a_1** and **6a_2** (Figure 4). The resulting complexation constants 37.3 M^−1^ for **6a_1** and 72.1 M^−1^ for **6a_2** showed the fairly good ability of our substances in enantioselective recognition of chiral guest molecules.

The presence of a trifluoroacetamide motif in several of our compounds led us to the idea of testing the anion-binding capacity as well. Indeed, compound **16** was shown to complex tetrabutylammonium acetate and benzoate in CDCl_3_ solution with corresponding constants of *K*_(Ac)_ = 23.6 M^−1^ and *K*_(Bz)_ = 29.9 M^−1^.

## 3. Materials and Methods

### 3.1. General Experimental Procedures

All chemicals were purchased from commercial sources and used without further purification. Solvents were dried and distilled using conventional methods. Melting points were measured on Heiztisch Mikroskop—Polytherm A (Wagner & Munz, Germany). NMR spectra were performed on Agilent 400-MR DDR2 (^1^H: 400 MHz, ^13^C: 100 MHz). Deuterated solvents used are indicated in each case. Chemical shifts (*δ*) are expressed in ppm and refer to the residual peak of the solvent or TMS as an internal standard; coupling constants (*J*) are in Hz. The mass analyses were performed using the ESI technique on a Q–TOF (Micromass) spectrometer. Elemental analyses were carried out on Perkin–Elmer 240, Elementar vario EL (Elementar, Germany) or Mitsubishi TOX–100 instruments. All samples were dried in the desiccator over P_2_O_5_ under vacuum (1 Torr) at 80 °C for 8 h. The IR spectra were measured on an FT–IR spectrometer Nicolet 740 or Bruker IFS66 spectrometers equipped with a heated Golden Gate Diamante ATR–Unit (SPECAC) in KBr. A total of 100 Scans for one spectrum were co–added at a spectral resolution of 4 cm^−1^. The courses of the reactions were monitored using TLC aluminium sheets with Silica gel 60 F254 (Merck). The column chromatography was performed on Silica gel 60 (Merck). HPLC was performed on Büchi Pure 850 FlashPrep chromatography instrument using Prontosil, 150 × 20 mm, 5 μm column.

### 3.2. Synthetic Procedures

#### 3.2.1. Quinoline Derivative **4a**

Calixarene **2** (0.122 g, 0.20 mmol) was dissolved in 5 mL of toluene at room temperature. Acetaldehyde (0.020 mL, 0.36 mmol) was added, and the resulting solution was stirred for 10 min. Then, trifluoroacetic acid (0.13 mL) was added and the colour of the solution immediately turned red. The reaction mixture was heated to 80 °C and stirred for 17 h. The solution was quenched by saturated NaHCO_3_ (5 mL). The organic phase was separated, washed with water (2 × 20 mL) and dried over magnesium sulphate. The solvent was removed under reduced pressure to yield a crude product, which was further purified by thin-layer chromatography on silica gel (cyclohexane:ethyl acetate 20:1, *v*/*v*) to give the title compound **4a** as a yellow amorphous solid (0.061 g, 46%), m.p. 173–176 °C.

^1^H NMR (CDCl_3_, 400 MHz, 298 K) *δ* 8.00 (d, 1H, *J* = 8.6 Hz, Ar-*H*), 7.51 (s, 1H, Ar-*H*), 7.20 (d, 1H, *J* = 8.2 Hz, Ar-*H*), 7.16–7.08 (m, 2H, Ar-*H*), 6.92 (t, 1H, *J* = 7.4 Hz, Ar-*H*), 6.22–6.05 (m, 5H, Ar-*H*), 5.96–5.89 (m, 1H, Ar-*H*), 5.03 (d, 1H, *J* = 13.3 Hz, Ar-*CH_2_*-Ar), 4.66 (d, 1H, *J* = 13.3 Hz, Ar-*CH_2_*-Ar), 4.52 (d, 1H, *J* = 13.3 Hz, Ar-*CH_2_*-Ar), 4.51 (d, 1H, *J* = 13.3 Hz, Ar-*CH_2_*-Ar), 4.35 (d, 1H, *J* = 13.3 Hz, Ar-*CH_2_*-Ar), 4.25–4.13 (m, 2H, O-*CH_2_*), 4.13–4.03 (m, 2H, O-*CH_2_*), 3.90–3.80 (m, 1H, O-*CH_2_*), 3.79–3.69 (m, 3H, O-*CH_2_*), 3.37 (d, 1H, *J* = 13.3 Hz, Ar-*CH_2_*-Ar), 3.24–3.14 (m, 2H, Ar-*CH_2_*-Ar), 2.76 (s, 3H, Ar-*CH_3_*), 2.11–1.87 (m, 8H, O-CH_2_-*CH_2_*), 1.21–1.09 (m, 6H, O-CH_2_-CH_2_-*CH_3_*), 0.99–0.87 (m, 6H, O-CH_2_-CH_2_-*CH_3_*) ppm. ^13^C NMR (CDCl_3_, 100 MHz, 298 K) *δ* 159.1, 158.1, 157.3, 155.3 (2×), 147.4, 137.4, 137.3, 137.0, 135.6, 134.4, 133.5, 133.0, 132.8, 132.4, 128.9, 128.8, 127.6, 127.4, 127.2, 126.9, 125.5, 122.9, 122.1, 122.0, 121.7, 119.8, 77.1, 76.9, 76.8, 76.5, 31.5, 31.1, 31.0, 26.9, 25.6, 23.6, 23.1, 23.0, 22.8, 10.9 (2×), 9.9 (2×) ppm. IR (KBr) *ν* 2961.1, 2931.8, 2874.3, 1590.4, 1454.9, 1383.1, 1245.0, 1193.7, 1005.9 cm^−1^. HRMS (ESI^+^) calcd for C_44_H_51_NO_4_ 658.3891 [M+H]^+^, 680.3710 [M+Na]^+^, found *m/z* 658.3892 [M+H]^+^ (100%), 680.3705 [M+Na]^+^ (10%).

#### 3.2.2. Quinoline Derivative **4b**

Calixarene **2** (0.101 g, 0.17 mmol) was dissolved in 5 mL of toluene at room temperature. Bromoacetaldehyde diethyl acetal (0.080 mL, 0.53 mmol) was added, and the resulting solution was stirred for 10 min. Then, trifluoroacetic acid (0.25 mL) was added and the colour of the solution immediately turned red. The reaction mixture was heated to 80 °C and stirred for 17 h. The solution was quenched by saturated NaHCO_3_ (5 mL). The organic phase was separated, washed with water (2 × 20 mL) and dried over magnesium sulphate. The solvent was removed under reduced pressure to yield a crude product, which was further purified by thin-layer chromatography on silica gel (cyclohexane:ethyl acetate 6:1, *v*/*v*) to give the title compound **5b** as a brown amorphous solid (0.061 g, 37%), m.p. 179–182 °C.

^1^H NMR (CDCl_3_, 400 MHz, 298 K) *δ* 8.24 (s, 1H, Ar-*H*), 7.41 (s, 1H, Ar-*H*), 6.98 (dd, 2H, *J* = 7.4, 1.2 Hz, Ar-*H*), 6.93 (dd, 1H, *J* = 7.4, 1.2 Hz, Ar-*H*), 6.76 (t, 1H, *J* = 7.4 Hz, Ar-*H*), 6.25–6.16 (m, 4H, Ar-*H*), 6.11 (dd, 1H, *J* = 6.3, 2.7 Hz, Ar-*H*), 6.02 (dd, 1H, *J* = 6.3, 2.7 Hz, Ar-*H*), 5.30 (s, 1H, CH_2_-*OH*), 4.89 (s, 2H, Ar-*CH_2_*-OH), 4.74 (d, 1H, *J* = 13.7 Hz, Ar-*CH_2_*-Ar), 4.65 (d, 1H, *J* = 13.3 Hz, Ar-*CH_2_*-Ar), 4.47 (d, 1H, *J* = 13.3 Hz, Ar-*CH_2_*-Ar), 4.46 (d, 1H, *J* = 13.3 Hz, Ar-*CH_2_*-Ar), 4.42 (d, 1H, *J* = 13.7 Hz, Ar-*CH_2_*-Ar), 4.15 (dd, 1H, *J* = 8.6, 7.0 Hz, O-*CH_2_*), 4.07–3.95 (m, 2H, O-*CH_2_*), 3.87–3.66 (m, 5H, O-*CH_2_*), 3.37 (d, 1H, *J* = 13.3 Hz, Ar-*CH_2_*-Ar), 3.16 (d, 1H, *J* = 13.3 Hz, Ar-*CH_2_*-Ar), 3.15 (d, 1H, *J* = 13.7 Hz, Ar-*CH_2_*-Ar), 2.06–1.96 (m, 3H, O-CH_2_-*CH_2_*), 1.95–1.84 (m, 5H, O-CH_2_-*CH_2_*), 1.10 (t, 6H, *J* = 7.4 Hz, O-CH_2_-CH_2_-*CH_3_*), 0.93 (t, 3H, *J* = 7.4 Hz, O-CH_2_-CH_2_-*CH_3_*), (t, 3H, *J* = 7.4 Hz, O-CH_2_-CH_2_-*CH_3_*) ppm. ^13^C NMR (CDCl_3_, 125 MHz, 298 K) *δ* 159.6, 157.7, 155.5 (2×), 154.2, 144.2, 139.2, 138.7, 136.9, 136.5, 134.1, 133.7, 133.5, 132.5, 132.2, 128.7 (2×), 128.0, 127.5, 127.4, 127.3, 125.3, 124.9, 122.1 (2×), 121.8, 113.2, 77.0 (2×), 76.9, 76.5, 63.7, 31.5, 31.1, 31.0, 23.7, 23.5, 23.4, 23.2, 23.1, 10.8 (2×), 10.00 (2×) ppm. IR (KBr) *ν* 2961.6, 2931.8, 2874.7, 1729.8, 1588.2, 1455.7, 1383.8, 1203.5, 1086.9 cm^−1^. HRMS (ESI^+^) calcd for C_44_H_50_NBrO_5_ 776.2744 [M+Na]^+^, 792.2484 [M+K]^+^, found *m/z* 776.2748 [M+Na]^+^ (100%), 792.2476 [M+K]^+^ (5%).

#### 3.2.3. Quinoline Derivative **6a**

Calixarene **3** (0.101 g, 0.14 mmol) was dissolved in 5 mL of toluene at room temperature. Acetaldehyde (0.020 mL, 0.36 mmol) was added, and the resulting solution was stirred for 10 min. Then, trifluoroacetic acid (0.11 mL) was added and the colour of the solution immediately turned red. The reaction mixture was heated to 80 °C and stirred for 15 h. The solution was quenched by saturated NaHCO_3_ (5 mL). The organic phase was separated, washed with water (2 × 20 mL) and dried over magnesium sulphate. The solvent was removed under reduced pressure to yield a crude product, which was further purified by thin-layer chromatography on silica gel (cyclohexane:ethyl acetate 3:1, *v*/*v*) to give the title compound **6a** as a brown amorphous solid (0.019 g, 18%), m.p. 170–173 °C.

^1^H NMR (CDCl_3_, 400 MHz, 298 K) *δ* 8.35 (d, 1H, *J* = 8.6 Hz, Ar-*H*), 7.79 (s, 1H, Ar-*H*), 7.26 (d, 1H, *J* = 9.0 Hz, Ar-*H*), 7.01 (dd, 1H, *J* = 7.4, 1.6 Hz, Ar-*H*), 6.94 (dd, 1H, *J* = 7.4, 1.2 Hz, Ar-*H*), 6.76 (t, 1H, *J* = 7.4 Hz, Ar-*H*), 6.22–6.13 (m, 5H, Ar-*H*), 5.94 (dd, 1H, *J* = 7.0, 1.6 Hz, Ar-*H*), 4.63 (d, 1H, *J* = 12.9 Hz, Ar-*CH_2_*-Ar), 4.60 (d, 1H, *J* = 13.7 Hz, Ar-*CH_2_*-Ar), 4.46 (d, 1H, *J* = 13.3 Hz, Ar-*CH_2_*-Ar), 4.46 (d, 1H, *J* = 13.3 Hz, Ar-*CH_2_*-Ar), 4.14 (dt, 1H, *J* = 10.6, 5.5 Hz, O-*CH_2_*), 4.05–3.95 (m, 3H, O-*CH_2_*), 3.92 (d, 1H, *J* = 14.1 Hz, Ar-*CH_2_*-Ar), 3.83–3.70 (m, 4H, O-*CH_2_*), 3.42 (d, 1H, *J* = 13.3 Hz, Ar-*CH_2_*-Ar), 3.19–3.13 (m, 2H, Ar-*CH_2_*-Ar), 2.75 (s, 3H, Ar-*CH_3_*), 2.07–1.86 (m, 8H, O-CH_2_-*CH_2_*), 1.11 (t, 3H, *J* = 7.4 Hz, O-CH_2_-CH_2_-*CH_3_*), 1.10 (t, 3H, *J* = 7.4 Hz, O-CH_2_-CH_2_-*CH_3_*), 0.96–0.89 (m, 6H, O-CH_2_-CH_2_-*CH_3_*) ppm. ^13^C NMR (CDCl_3_, 100 MHz, 298 K) *δ* 157.7, 156.1, 155.6, 155.5, 155.4 (2×), 136.8, 136.7 (2×), 133.7 (2×), 133.2, 132.3, 130.3, 128.8, 128.6 (2×), 127.7 (2×), 127.6, 126.8, 125.8, 122.1 (3×), 121.7, 121.0, 76.9 (3×), 76.4, 31.7, 31.1, 30.9, 24.2, 23.5 (2×), 23.4, 23.1, 23.0, 10.8, 10.7, 10.0, 9.9 ppm. IR (KBr) *ν* 2960.5, 2920.4, 2874.1, 1455.4, 1383.7, 1208.3, 1086.9 cm^−1^. HRMS (ESI^+^) calcd for C_44_H_51_NO_4_ 658.3891 [M+H]^+^, 696.3450 [M+Na]^+^, found *m/z* 658.3894 [M+H]^+^ (100%), 696.3443 [M+Na]^+^ (3%).

#### 3.2.4. Bis-Quinoline Derivative **14**

Calixarene **7** (0.104 g, 0.17 mmol) was dissolved in 5 mL of toluene at room temperature. Acetaldehyde (0.040 mL, 0.71 mmol) was added, and the resulting solution was stirred for 10 min. Then, trifluoroacetic acid (0.22 mL) was added and the colour of the solution immediately turned red. The reaction mixture was heated to 80 °C and stirred for 15 h. The solution was quenched by saturated NaHCO_3_ (5 mL). The organic phase was separated, washed with water (2 × 20 mL) and dried over magnesium sulphate. The solvent was removed under reduced pressure to yield a crude product which was further purified by thin-layer chromatography on silica gel (cyclohexane:ethyl acetate 6:1, *v*/*v*) to give the title compound **14** as a yellow amorphous solid (0.061 g, 37%), m.p. 185–188 °C.

^1^H NMR (CDCl_3_, 400 MHz, 298 K) *δ* 8.00 (d, 2H, *J* = 8.2 Hz, Ar-*H*), 7.52 (s, 2H, Ar-*H*), 7.20 (d, 2H, *J* = 8.2 Hz, Ar-*H*), 6.21–6.05 (m, 2H, Ar-*H*), 6.03–5.95 (m, 2H, Ar-*H*), 5.84 (dd, 2H, *J* = 6.7, 2.0 Hz, Ar-*H*), 5.04 (d, 2H, *J* = 13.3 Hz, Ar-*CH_2_*-Ar), 4.67 (d, 2H, *J* = 13.3 Hz, Ar-*CH_2_*-Ar), 4.35 (d, 2H, *J* = 13.3 Hz, Ar-*CH_2_*-Ar), 4.25–4.16 (m, 2H, O-*CH_2_*), 3.90–3.68 (m, 6H, O-*CH_2_*), 3.38 (d, 2H, *J* = 13.3 Hz, Ar-*CH_2_*-Ar), 2.75 (s, 6H, Ar-*CH_3_*), 2.13–1.85 (m, 8H, O-CH_2_-*CH_2_*), 1.17 (t, 6H, *J* = 7.4 Hz, O-CH_2_-CH_2_*-CH_3_*), 0.91 (t, 6H, *J* = 7.4 Hz, O-CH_2_-CH_2_*-CH_3_*) ppm. ^13^C NMR (CDCl_3_, 100 MHz, 298 K) *δ* 159.2, 157.3, 155.4, 147.4, 137.6, 135.6, 134.7, 133.0, 131.8, 127.5, 126.7, 125.5, 122.9, 122.1, 119.8, 76.9 (2×), 31.5, 25.6, 23.6, 23.1, 22.6, 10.9, 9.9 ppm. IR (KBr) *ν* 2960.1, 2921.7, 2874.2, 1602.4, 1492.0, 1453.8, 1382.6, 1184.0, 1085.3 cm^−1^. HRMS (ESI^+^) calcd for C_48_H_54_N_2_O_4_ 723.4156 [M+H]^+^, 745.3976 [M+Na]^+^, found *m/z* 723.4156 [M+H]^+^ (100%), 745.3972 [M+Na]^+^ (25%).

#### 3.2.5. Quinoline Derivatives **15a** and **15b**

Calixarene **8** (0.119 g, 0.19 mmol) was dissolved in 5 mL of toluene at room temperature. Acetaldehyde (0.040 mL, 0.71 mmol) was added, and the resulting solution was stirred for 10 min. Then, trifluoroacetic acid (0.25 mL) was added and the colour of the solution immediately turned red. The reaction mixture was heated to 80 °C and stirred for 17 h. The solution was quenched by saturated NaHCO_3_ (5 mL). The organic phase was separated, washed with water (2 × 20 mL) and dried over magnesium sulphate. The solvent was removed under reduced pressure to yield a crude product which was further purified by thin-layer chromatography on silica gel (cyclohexane:ethyl acetate 4:1, *v*/*v*) to give an inseparable mixture of title compounds **15a** and **15b** as a yellow amorphous solid (0.020 g, 14%), m.p. 152–155 °C.

^1^H NMR (CDCl_3_, 400 MHz, 298 K) *δ* 8.39 (t, 2H, *J* = 9.0 Hz, Ar-*H*), 7.98 (d, 2H, *J* = 8.6 Hz, Ar-*H*), 7.87 (s, 2H, Ar-*H*), 7.51 (s, 2H, Ar-*H*), 7.29 (d, 2H, *J* = 9.0 Hz, Ar-*H*), 7.19 (d, 2H, *J* = 8.2 Hz, Ar-*H*), 6.09–5.95 (m, 7H, Ar-*H*), 5.93 (t, 1H, *J* = 7.4 Hz, Ar-*H*), 5.86 (dd, 1H, *J* = 7.4, 1.6 Hz, Ar-*H*), 5.81 (dd, 1H, *J* = 7.4, 1.6 Hz, Ar-*H*), 5.78–5.71 (m, 2H, Ar-*H*), 5.03 (d, 1H, *J* = 13.3 Hz, Ar-*CH_2_*-Ar), 5.02 (d, 1H, *J* = 13.7 Hz, Ar-*CH_2_*-Ar), 4.70–4.55 (m, 4H, Ar-*CH_2_*-Ar), 4.33 (d, 1H, *J* = 12.9 Hz, Ar-*CH*_2_-Ar), 4.31 (d, 1H, *J* = 13.3 Hz, Ar-*CH_2_*-Ar), 4.26–4.00 (m, 8H, O-*CH_2_*), 3.94 (d, 1H, *J* = 14.1 Hz, Ar-*CH_2_*-Ar), 3.94 (d, 1H, *J* = 13.3 Hz, Ar-*CH_2_*-Ar), 3.87–3.68 (m, 8H, O-*CH_2_*), 3.45 (d, 1H, *J* = 13.3 Hz, Ar-*CH_2_*-Ar), 3.44 (d, 1H, *J* = 12.9 Hz, Ar-*CH_2_*-Ar), 3.36 (d, 1H, *J* = 13.3 Hz, Ar-*CH_2_*-Ar), 3.35 (d, 1H, *J* = 13.3 Hz, Ar-*CH_2_*-Ar), 2.76 (s, 3H, Ar-*CH_3_*), 2.73 (s, 3H, Ar-*CH_3_*), 2.14–1.83 (m, 16H, O-CH_2_-*CH_2_*), 1.19–1.10 (m, 12H, O-CH_2_-CH_2_-*CH_3_*), 0.97–0.84 (m, 12H, O-CH_2_-CH_2_-*CH_3_*) ppm. HRMS (ESI^+^) calcd for C_48_H_54_N_2_O_4_ 723.4156 [M+H]^+^, 745.3976 [M+Na]^+^, found *m/z* 723.4162 [M+H]^+^ (100%), 745.3974 [M+Na]^+^ (20%).

#### 3.2.6. Quinoline Derivative **15c**

Calixarene **15c** was isolated from the same reaction mixture as compounds **15a** and **15b**. The product was obtained as a yellow amorphous solid (0.014 g, 10%), m.p. 163–166 °C.

^1^H NMR (CDCl_3_, 500 MHz, 298 K) *δ* 7.98 (d, 1H, *J* = 8.2 Hz, Ar-*H*), 7.48 (s, 1H, Ar-*H*), 7.18 (d, 1H, *J* = 8.2 Hz, Ar-*H*), 6.43 (s, 1H, Ar-*NH*-CH_2_), 6.26–6.01 (m, 5H, Ar-*H*), 5.89 (d, 1H, *J* = 7.0 Hz, Ar-*H*), 4.99 (d, 1H, *J* = 13.3 Hz, Ar-*CH_2_*-Ar), 4.62 (d, 1H, *J* = 13.3 Hz, Ar-*CH_2_*-Ar), 4.43 (d, 1H, *J* = 13.3 Hz, Ar-*CH_2_*-Ar), 4.42 (d, 1H, *J* = 12.9 Hz, Ar-*CH_2_*-Ar), 4.21–4.08 (m, 2H, O-*CH_2_*), 3.98–3.89 (m, 2H, O-*CH_2_*), 3.83–3.65 (m, 4H, O-*CH_2_*), 3.34 (d, 1H, *J* = 13.3 Hz, Ar-*CH_2_*-Ar), 3.16 (q, 2H, *J* = 7.0 Hz, NH-*CH_2_*-CH_3_), 3.05 (d, 2H, *J* = 13.3 Hz, Ar-*CH_2_*-Ar), 2.74 (s, 3H, Ar-*CH_3_*), 2.10–1.83 (m, 8H, O-CH_2_-*CH_2_*), 1.29 (t, 3H, *J* = 7.0 Hz, NH-CH_2_-*CH_3_*), 1.12 (t, 3H, *J* = 7.4 Hz, O-CH_2_-CH_2_-*CH_3_*), 1.11 (t, 3H, *J* = 7.4 Hz, O-CH_2_-CH_2_-*CH_3_*), 0.90 (t, 3H, *J* = 7.4 Hz, O-CH_2_-CH_2_-*CH_3_*), 0.87 (t, 3H, *J* = 7.4 Hz, O-CH_2_-CH_2_-*CH_3_*) ppm. ^13^C NMR (CDCl_3_, 125 MHz, 298 K) *δ* 157.2, 155.3, 155.2, 137.7, 137.3, 135.6, 134.3, 133.5, 132.8, 132.3, 127.6, 127.3, 127.1, 126.8, 125.4, 122.8, 122.0, 121.9, 119.7, 77.0, 76.9, 76.7, 76.6, 31.5, 31.2, 31.1, 29.7, 25.5, 23.6, 23.5, 23.0, 22.9, 15.0, 10.9, 10.8, 9.9 (2×) ppm. IR (KBr) *ν* 2959.7, 1219.1, 173.9, 1958.3, 1604.5, 1454.2, 1383.3, 1087.7 cm^−1^. HRMS (ESI^+^) calcd for C_46_H_56_N_2_O_4_ 701.4313 [M+H]^+^, 723.4132 [M+Na]^+^, found *m/z* 701.4318 [M+H]^+^ (100%), 723.4134 [M+Na]^+^ (75%).

#### 3.2.7. Quinoline Derivative **16**

Calixarene **12** (0.101 g, 0.14 mmol) was dissolved in 5 mL of toluene at room temperature. Acetaldehyde (0.020 mL, 0.36 mmol) was added, and the resulting solution was stirred for 10 min. Then, trifluoroacetic acid (0.09 mL) was added and the colour of the solution immediately turned red. The reaction mixture was heated to 80 °C and stirred for 17 h. The solution was quenched by saturated NaHCO_3_ (5 mL). The organic phase was separated, washed with water (2 × 20 mL) and dried over magnesium sulphate. The solvent was removed under reduced pressure to yield crude product, which was further purified by thin-layer chromatography on silica gel (cyclohexane:ethyl acetate 4:1, *v*/*v*) to give the title compound **16** as a yellow amorphous solid (0.051 g, 47%), m.p. 163–166 °C.

^1^H NMR (CDCl_3_, 400 MHz, 298 K) *δ* 7.92 (d, 1H, *J* = 8.2 Hz, Ar-*H*), 7.72 (br s, 1H, Ar-*H*), 7.40 (s, 1H, Ar-*H*), 7.14 (d, 1H, *J* = 8.2 Hz, Ar-*H*), 7.09 (br s, 1H, Ar-*H*), 6.32–6.07 (m, 6H, Ar-*H*), 4.92 (d, 1H, *J* = 12.9 Hz, Ar-*CH_2_*-Ar), 4.62 (d, 1H, *J* = 13.3 Hz, Ar-*CH_2_*-Ar), 4.53–4.42 (m, 1H, Ar-*CH_2_*-Ar), 4.34 (d, 1H, *J* = 12.9 Hz, Ar-*CH_2_*-Ar), 4.17–3.93 (m, 4H, O-*CH_2_*), 3.89–3.79 (m, 1H, O-*CH_2_*), 3.79–3.67 (m, 3H, O-*CH_2_*), 3.35 (d, 1H, *J* = 13.3 Hz, Ar-*CH_2_*-Ar), 3.17 (d, 1H, *J* = 13.3 Hz, Ar-*CH_2_*-Ar), 3.15 (d, 1H, *J* = 13.3 Hz, Ar-*CH_2_*-Ar), 2.72 (s, 3H, Ar-*CH_3_*), 2.07–1.82 (m, 8H, O-CH_2_-*CH_2_*), 1.18–1.03 (m, 6H, O-CH_2_-CH_2_-*CH_3_*), 1.00–0.85 (m, 6H, O-CH_2_-CH_2_-*CH_3_*) ppm. ^13^C NMR (CDCl_3_, 100 MHz, 298 K) *δ* 158.8, 158.7, 157.1, 156.0, 155.5 (2×), 137.9, 137.5, 137.2, 136.9, 135.4, 134.8, 134.5, 133.0 (2×), 132.2, 128.6, 128.3, 127.7, 127.6, 126.9, 125.6, 122.9, 122.2, 121.9, 120.8 (2×), 119.7, 117.3, 77.0, 76.9 (2×), 76.1, 31.3, 31.0 (2×), 25.5, 23.5 (3×), 23.1, 23.0, 10.7, 10.0 (2×), 9.6 ppm. IR (KBr) *ν* 3305.1, 2962.0, 2933.1, 2875.5, 1710.2, 1605.4, 1455.9, 1183.1, 758.4 cm^−1^. HRMS (ESI^+^) calcd for C_46_H_51_F_3_N_2_O_5_ 769.3823 [M+H]^+^, 791.3642 [M+Na]^+^, 807.3382 [M+K]^+^, found *m/z* 769.3827 [M+H]^+^ (100%), 791.3638 [M+Na]^+^ (30%), 807.3375 [M+K]^+^ (18%).

#### 3.2.8. Quinoline Derivative **17a**

Calixarene **13** (0.124 g, 0.17 mmol) was dissolved in 5 mL of toluene at room temperature. Acetaldehyde (0.020 mL, 0.36 mmol) was added, and the resulting solution was stirred for 10 min. Then, trifluoroacetic acid (0.11 mL) was added and the colour of the solution immediately turned red. The reaction mixture was heated to 80 °C and stirred for 17 h. The solution was quenched by saturated NaHCO_3_ (5 mL). The organic phase was separated, washed with water (2 × 20 mL) and dried over magnesium sulphate. The solvent was removed under reduced pressure to yield a crude product, which was further purified by preparative HPLC to give the title compounds 17a as a yellow amorphous solid (0.023 g, 17%), m.p. 120–123 °C. In the ^13^C NMR spectrum, the number of signals does not correspond to the number of carbons due to the low intensity of certain signals.

^1^H NMR (CDCl_3_, 500 MHz, 298 K) *δ* 7.73 (s, 1H, Ar-*H*), 7.23–6.73 (m, 8H, Ar-*H*, Ar-*NH*-COCF_3_), 6.67–6.53 (m, 1H, Ar-*H*), 6.26–6.05 (m, 2H, Ar-*H*), 4.84 (d, 1H, *J* = 14.0 Hz, Ar-*CH_2_*-Ar), 4.61 (d, 1H, *J* = 13.5 Hz, Ar-*CH_2_*-Ar), 4.47 (d, 1H, *J* = 14.9 Hz, Ar-*CH_2_*-Ar), 4.40 (d, 1H, *J* = 13.5 Hz, Ar-*CH_2_*-Ar), 4.01–3.90 (m, 4H, O-*CH_2_*), 3.80 (d, 1H, *J* = 14.6 Hz, Ar-*CH_2_*-Ar), 3.77–3.59 (m, 4H, O-*CH_2_*), 3.37 (d, 1H, *J* = 13.5 Hz, Ar-*CH_2_*-Ar), 3.19–3.05 (m, 2H, Ar-*CH_2_*-Ar), 2.52 (s, 3H, Ar-*CH_3_*), 1.98–1.74 (m, 8H, O-CH_2_-*CH_2_*), 1.10 (t, 3H, *J* = 7.1 Hz, O-CH_2_-CH_2_-*CH_3_*), 1.04 (t, 3H, *J* = 7.4 Hz, O-CH_2_-CH_2_-*CH_3_*), 0.92 (t, 3H, *J* = 7.1 Hz, O-CH_2_-CH_2_-*CH_3_*), 1.06–0.81 (m, 3H, O-CH_2_-CH_2_-*CH_3_*) ppm. ^13^C NMR (CDCl_3_, 125 MHz, 298 K) *δ* 155.7, 145.1, 132.4, 129.8, 129.1, 129.0, 128.6, 128.4, 122.6, 122.3, 121.9, 118.2, 114.9, 114.1, 77.2, 77.1, 76.9, 76.3, 31.1, 30.9, 29.7, 26.9, 24.6, 23.5, 23.4, 23.1, 21.9, 10.7 (2×), 10.0, 9.6. ppm. IR (KBr) *ν* 3360.6, 2961.8, 2920.2, 2875.8, 1729.1, 1454.9, 1203.2, 1157.5 cm^−1^. HRMS (ESI^+^) calcd for C_46_H_51_F_3_N_2_O_5_ 769.3823 [M+H]^+^, 791.3642 [M+Na]^+^, 807.3382 [M+K]^+^, found *m/z* 769.3827 [M+H]^+^ (100%), 791.3638 [M+Na]^+^ (30%), 807.3375 [M+K]^+^ (15%).

#### 3.2.9. Quinoline Derivative **17b**

Calixarene **17b** was isolated from the same reaction mixture as compound **17a**. This product was isolated as a yellow amorphous solid (0.020 g, 15%), m.p. 122–125 °C. In the ^13^C NMR spectrum, the number of signals does not correspond to the number of carbons due to the low intensity of certain signals.

^1^H NMR (CDCl_3_, 400 MHz, 298 K) *δ* 8.03 (s, 1H, Ar-*H*), 7.77 (s, 1H, Ar-*NH*-COCF_3_), 7.06–6.99 (m, 2H, Ar-*H*), 6.83–6.48 (m, 6H, Ar-*H*), 6.29–5.96 (m, 2H, Ar-*H*), 4.73 (d, 1H, *J* = 14.1 Hz, Ar-*CH_2_*-Ar), 4.60 (d, 1H, *J* = 13.7 Hz, Ar-*CH_2_*-Ar), 4.44 (d, 1H, *J* = 14.4 Hz, Ar-*CH_2_*-Ar), 4.38 (d, 1H, *J* = 13.8 Hz, Ar-*CH_2_*-Ar), 4.04–3.90 (m, 4H, O-*CH_2_*), 3.88–3.72 (m, 5H, O-*CH_2_*, Ar-*CH_2_*-Ar), 3.39 (d, 1H, *J* = 13.8 Hz, Ar-*CH_2_*-Ar), 3.24 (d, 1H, *J* = 14.5 Hz, Ar-*CH_2_*-Ar), 3.10 (d, 1H, *J* = 13.8 Hz, Ar-*CH_2_*-Ar), 2.60 (s, 3H, Ar-*CH_3_*), 2.02–1.77 (m, 8H, O-CH_2_-*CH_2_*), 1.08–0.92 (m, 12H, O-CH_2_-CH_2_-*CH_3_*) ppm. ^13^C NMR (CDCl_3_, 100 MHz, 298 K) *δ* 156.0, 144.9, 140.2, 132.8, 130.4, 128.9, 128.3, 127.9, 124.8, 122.4, 121.9, 120.2, 118.7, 117.2, 77.0, 76.8 (2×), 31.7, 31.1, 29.7, 26.9, 24.8, 23.3, 23.2, 22.9, 10.5, 10.4, 10.2 (2×) ppm. IR (KBr) *ν* 3352.4, 2961.7, 2920.3, 2875.9, 1728.4, 1454.4, 1203.9, 1157.9 cm^−1^. HRMS (ESI^+^) calcd for C_46_H_51_F_3_N_2_O_5_ 769.3823 [M+H]^+^, 791.3642 [M+Na]^+^, 807.3382 [M+K]^+^, found *m/z* 769.3815 [M+H]^+^ (100%), 791.3635 [M+Na]^+^ (8%), 807.3375 [M+K]^+^ (20%).

### 3.3. Chiral Separation

Chiral separation of **6a** was performed using a Büchi Pure 850 FlashPrep chromatography instrument consisting of a binary pump module, UV-vis detector, column manager and fraction collector. The suitable conditions allowing for efficient enantioseparation were first proven on the analytical scale using chiral polysaccharide column ChiralArt Amylose-SA (250 × 4.6 mm ID, 5 μm). In preparative mode, a polysaccharide column ChiralArt Amylose-SA (250 × 20 mm ID, 5 μm) was employed using cyclohexane/DCM: 92/8 *v/v* as a mobile phase.

### 3.4. NMR Titrations

In each case, calixarene was dissolved in a specified amount of CDCl_3_ or C_2_D_2_Cl_4_ and 0.5 mL of calixarene solution was put in an NMR tube. A specific amount of guest was added to the calixarene solution (0.6 mL), and the aliquots of guest were gradually added to the NMR tube to achieve different calixarene/guest ratios, ensuring constant calixarene concentration during the experiment. The complexation constants were determined by analyzing CIS (complexation induced chemical shifts) of calixarene protons using a nonlinear curve-fitting procedure (program BindFit) [30].

### 3.5. X-ray Measurements

Crystallographic data for **16.** M = 800.94 g·mol^−1^, triclinic system, space group *P-1*, *a* = 15.52150 (2) Å, *b* = 18.08924 (3) Å, *c* = 18.11720 (2) Å, *α* = 77.920 (3)°, *β* = 71.014 (2)°, *γ* = 64.684 (2)° Z = 4, V = 4333.46 (9) Å^3^, *D_c_* = 1.228 g·cm^−3^, *μ*(Cu-Kα) = 0.73 mm^−1^, crystal dimensions of 0.41 × 0.29 × 0.26 mm. Data were collected at 200 (2) K on a Bruker D8 Venture Photon CMOS diffractometer with Incoatec microfocus sealed tube Cu-Kα radiation. The structure was solved by charge flipping methods [31] and anisotropically refined by full matrix least squares on F squared using the CRYSTALS [32] to final value R = 0.085 and wR = 0.242 using 15,813 independent reflections (*θ_max_* = 68.4°), 1370 parameters and 481 restrains. The hydrogen atoms bonded to carbon atoms were placed in calculated positions refined with riding constrains, while hydrogen atoms bonded to oxygen and nitrogen were refined using soft restraints. The disordered functional group positions were found in difference electron density maps and refined with restrained geometry. MCE [33] was used for visualization of electron density maps. The occupancy of the disordered functional group was fully constrained. The structure was deposited into the Cambridge Structural Database under the number CCDC 2215763.

## 4. Conclusions

In summary, the construction of inherently chiral calixarenes by the intramolecular cyclization of suitable intermediates suffers from a limited number of suitable substrates for these reactions. Here, we report on an easy way to prepare one class of such compounds: calixquinolines, which can be obtained by the reaction of aldehydes with easily accessible aminocalix[4]arenes under acidic conditions (Doebner–Miller reaction). The synthetic procedure represents a very straightforward approach to the inherently chiral macrocyclic systems. The complexation studies revealed the ability of these compounds to complex quaternary ammonium salts with different stoichiometries depending on the guest molecules; moreover, we demonstrated their ability to carry out the enantioselective complexation of chiral *N*-methylammonium salts.

## Data Availability

All experimental data are provided in the Supplementary information.

## Supplementary Materials

The following supporting information can be downloaded at: https://www.mdpi.com/article/10.3390/molecules27238545/s1. Spectral characterization of all new compounds (^1^H NMR, ^13^C NMR, HRMS, IR) and the NMR complexation studies.
