# Supplementary material for: The Formation of Inherently Chiral Calix[4]quinolines by Doebner–Miller Reaction of Aldehydes and Aminocalixarenes
Source: Molecules. 2022 Dec 4;27(23):8545. doi: 10.3390/molecules27238545 (PMC9736694; doi:10.3390/molecules27238545)
Supplement: Supplementary file 1 [file molecules-27-08545-s001.zip › molecules-2035672-supplementary.pdf]

Supporting Information

The Formation of Inherently Chiral Calix[4]quinolines by Doebner–Miller reaction of Aldehydes and Aminocalixarenes

Martin Tlustý,[†] Václav Eigner[§] , Hana Dvořáková[ʃ] and Pavel Lhoták[†*]

[†]Department of Organic Chemistry, University of Chemistry and Technology, Prague (UCTP), Technická 5, 166 28 Prague 6, Czech Republic

[§]Department of Solid State Chemistry, UCTP, Technická 5, 166 28 Prague 6, Czech Republic

[ʃ]Laboratory of NMR spectroscopy, UCTP, Technická 5, 166 28 Prague 6, Czech Republic

Table of Contents

1. Spectral characterization of compounds 2

2. Crystallographic data 19

3. Chiral separation 21

4. Titration experiments 22

5. VT ^{1}H NMR experiments 29

1. Spectral characterization of compounds

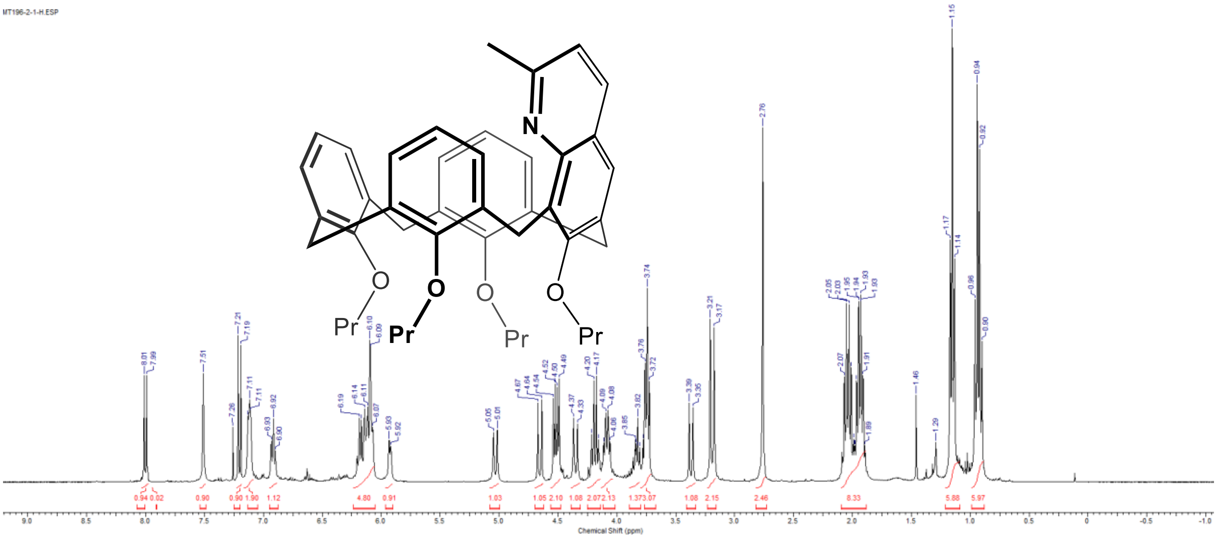

Figure S1: ^{1}H NMR of compound **4a** ($CDCl_3$, 400 MHz).

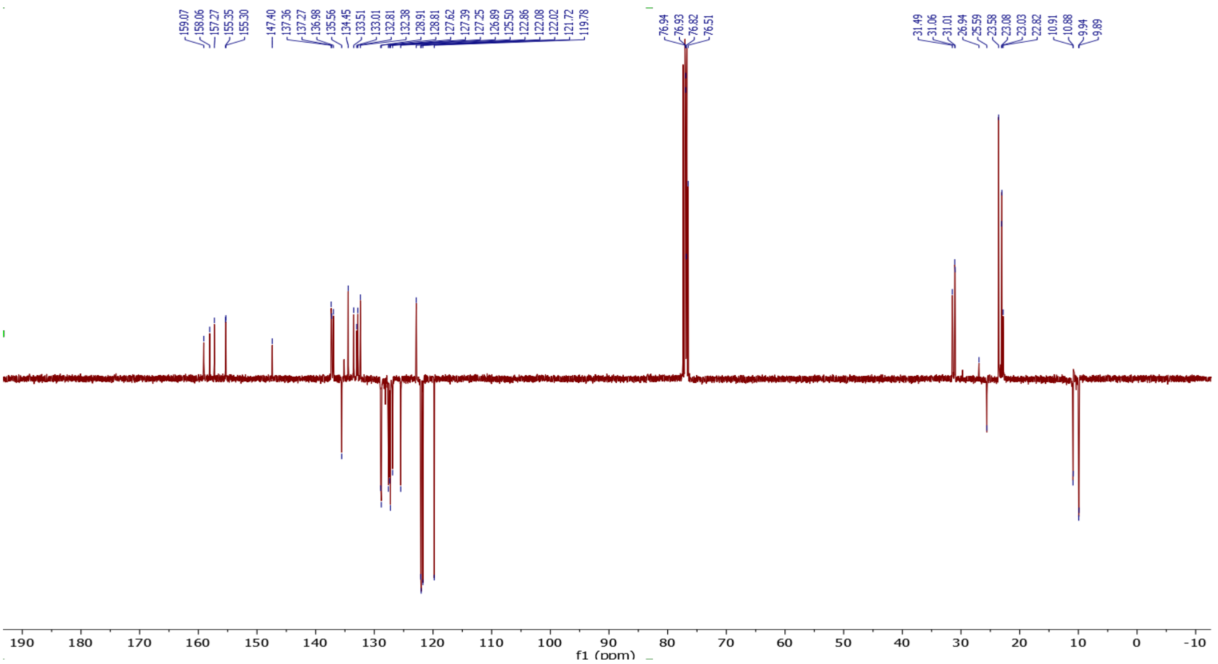

Figure S2: ^{13}C(APT) NMR of compound **4a** ($CDCl_3$, 100 MHz).

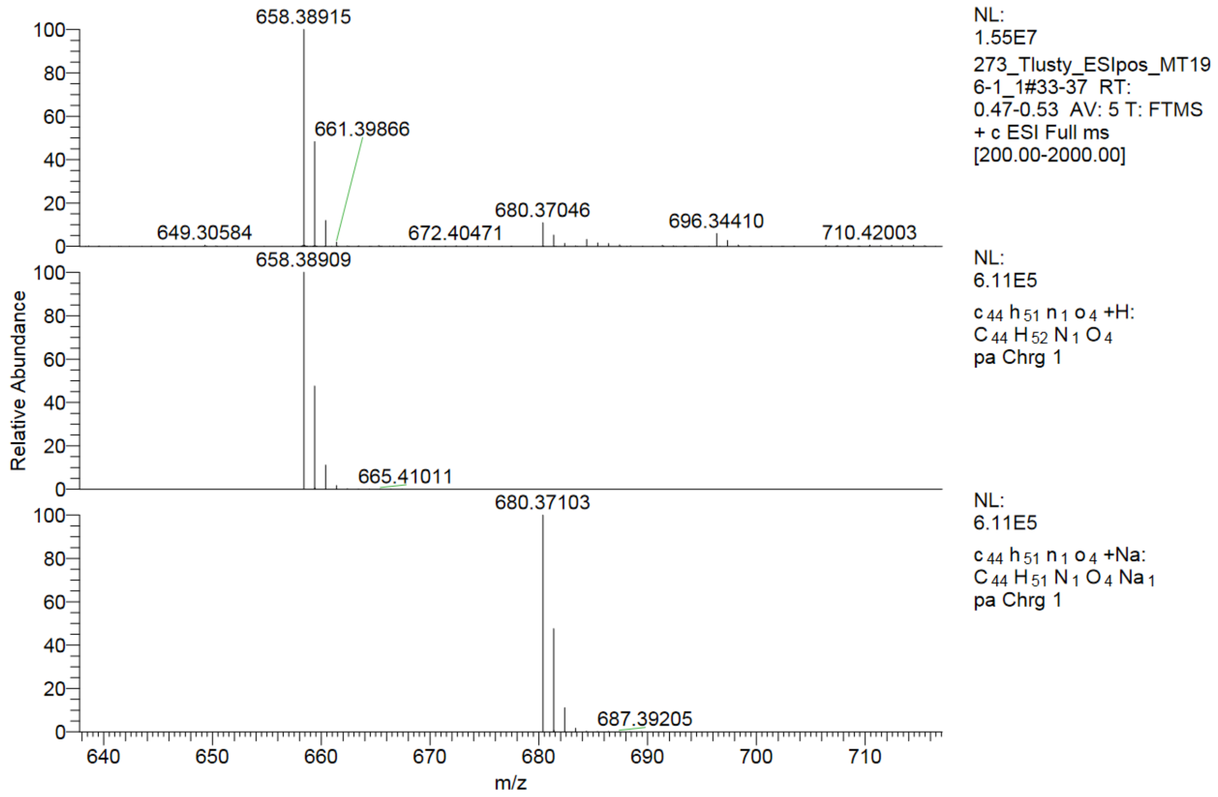

Figure S3: HRMS of compound **4a** (ESI⁺).

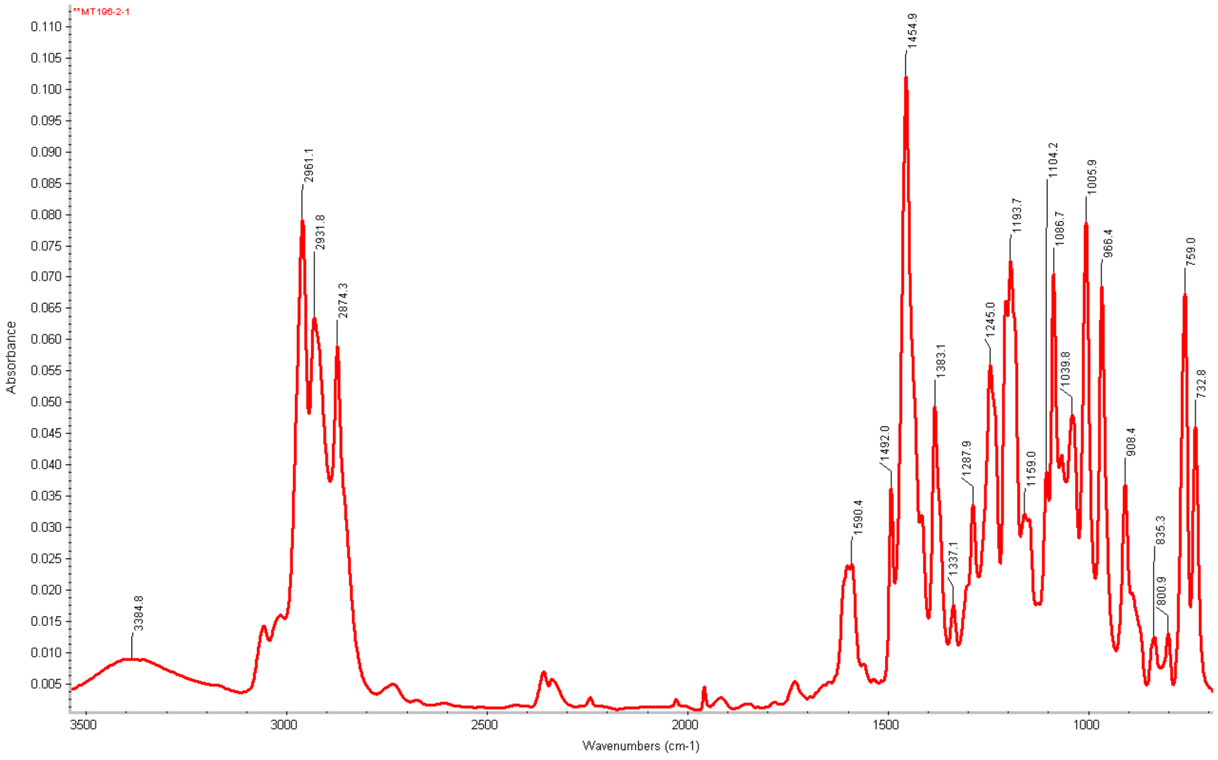

Figure S4: IR of compound **4a** (KBr).

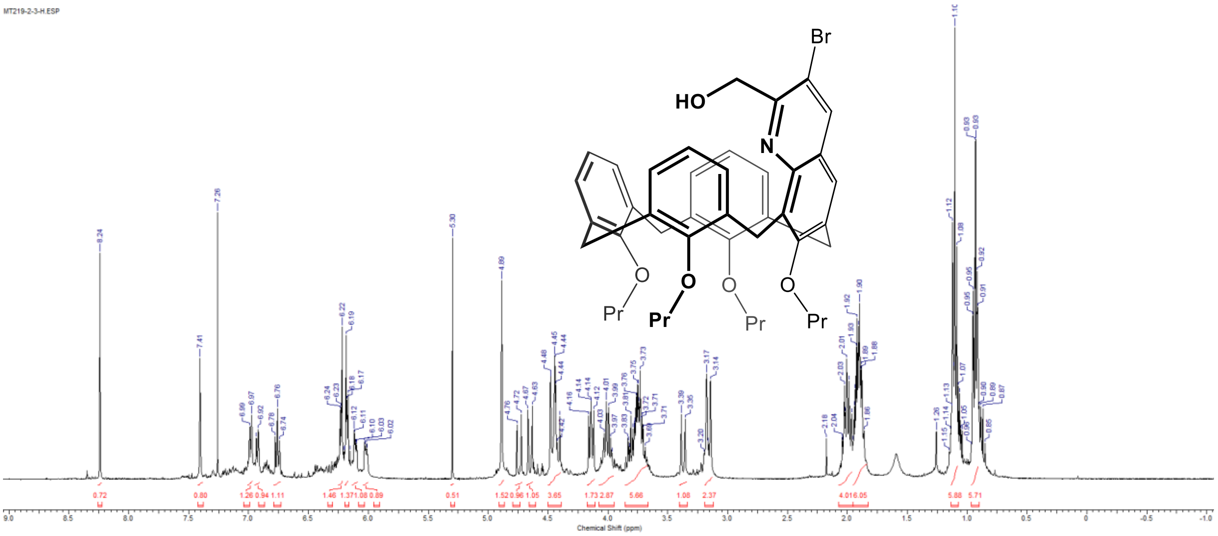

Figure S5: ^{1}H NMR of compound **4b** ($CDCl_3$, 400 MHz).

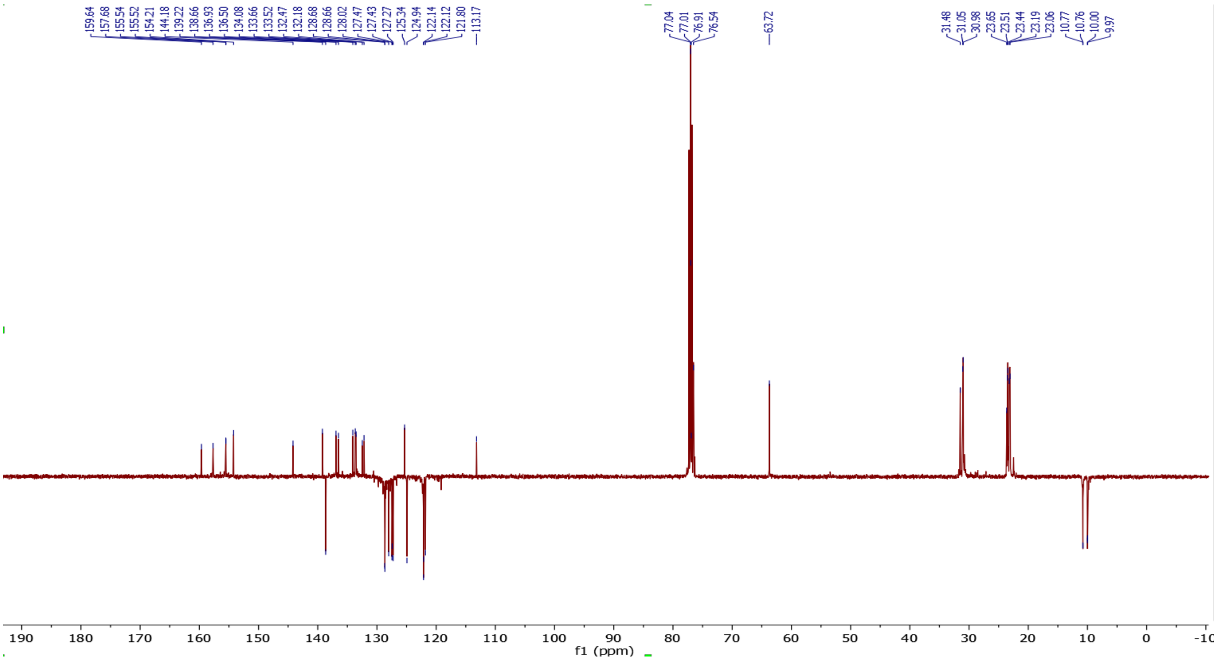

Figure S6: ^{13}C(APT) NMR of compound **4b** ($CDCl_3$, 100 MHz).

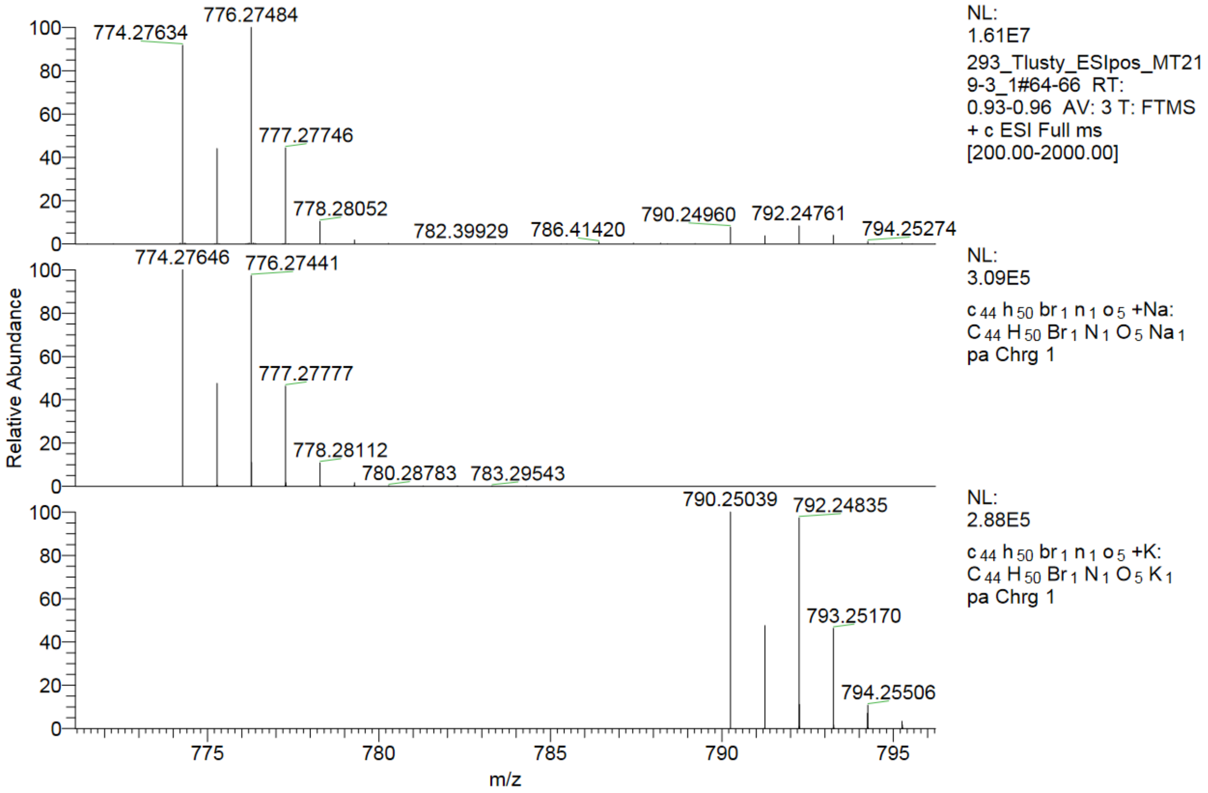

Figure S7: HRMS of compound **4b** (ESI+).

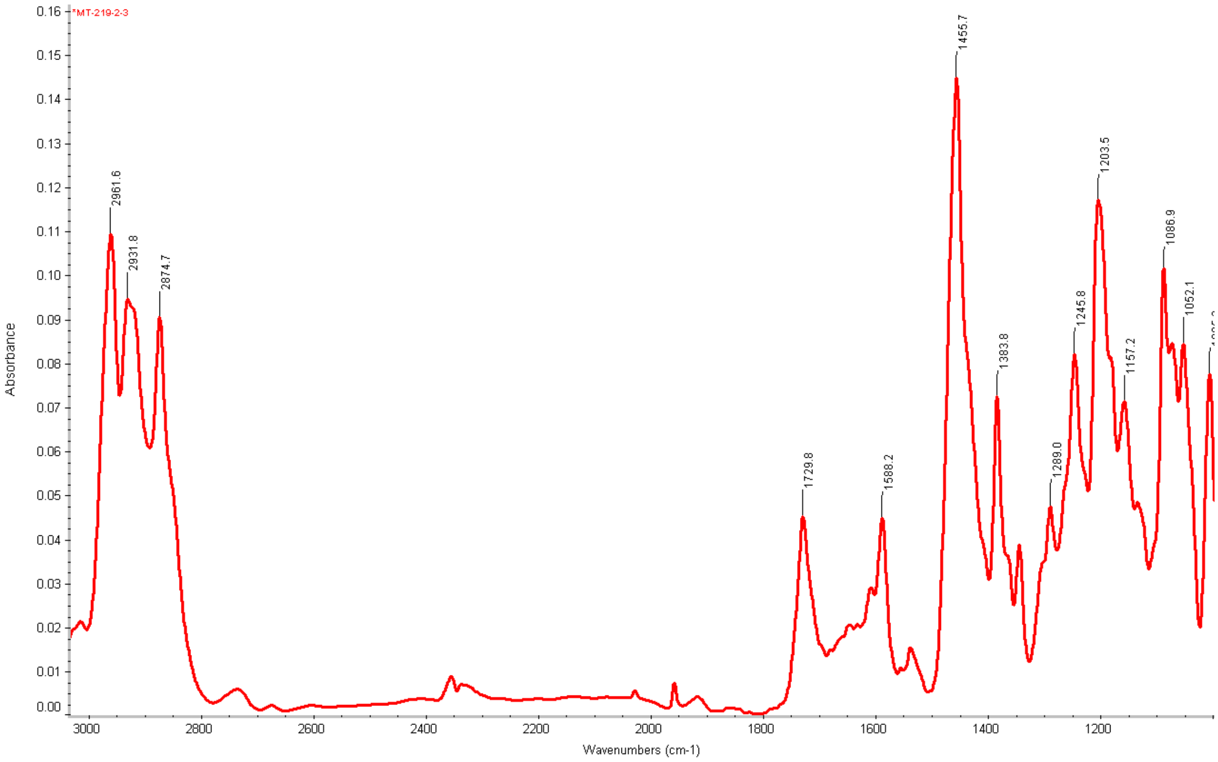

Figure S8: IR of compound **4b** (KBr).

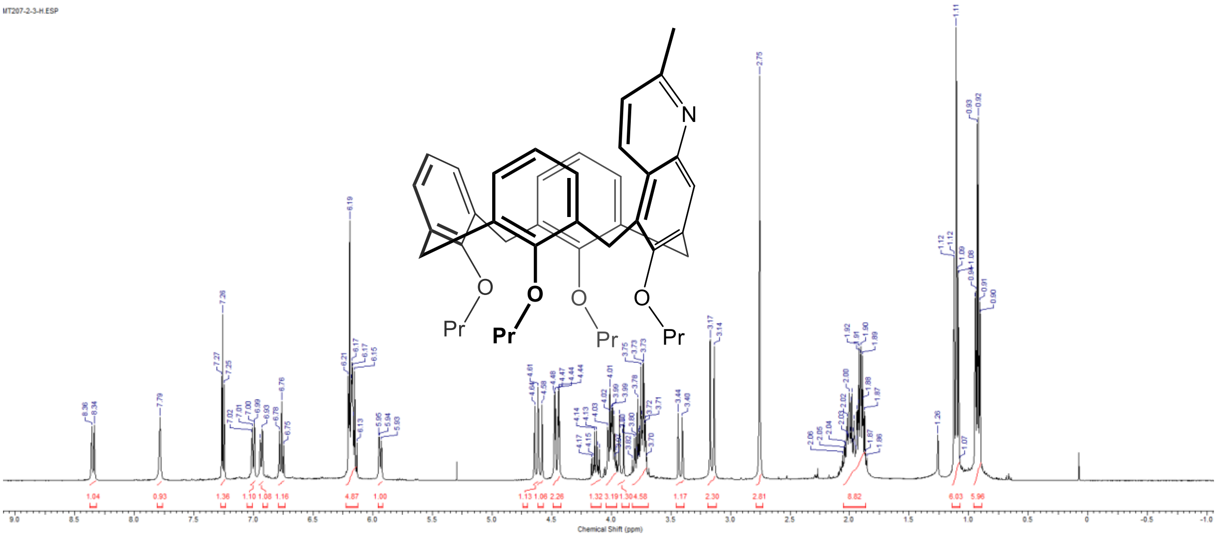

Figure S9: ¹H NMR of compound **6a** ($CDCl_3$, 400 MHz).

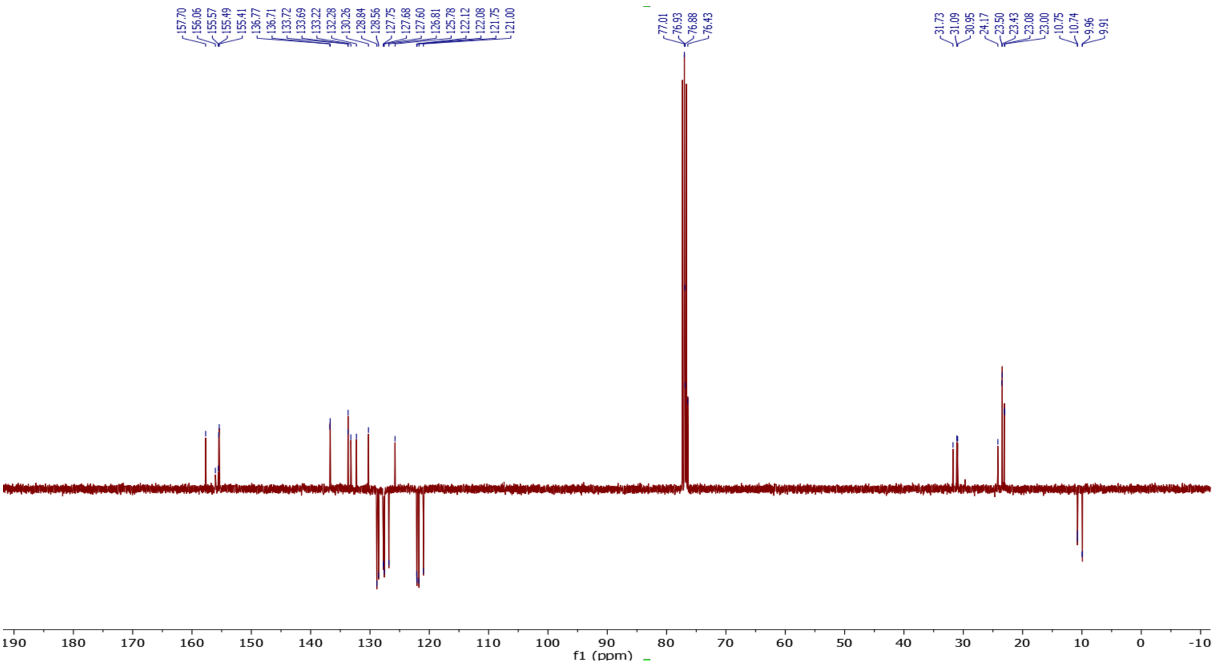

Figure S10: ¹³C(APT) NMR of compound **6a** ($CDCl_3$, 100 MHz).

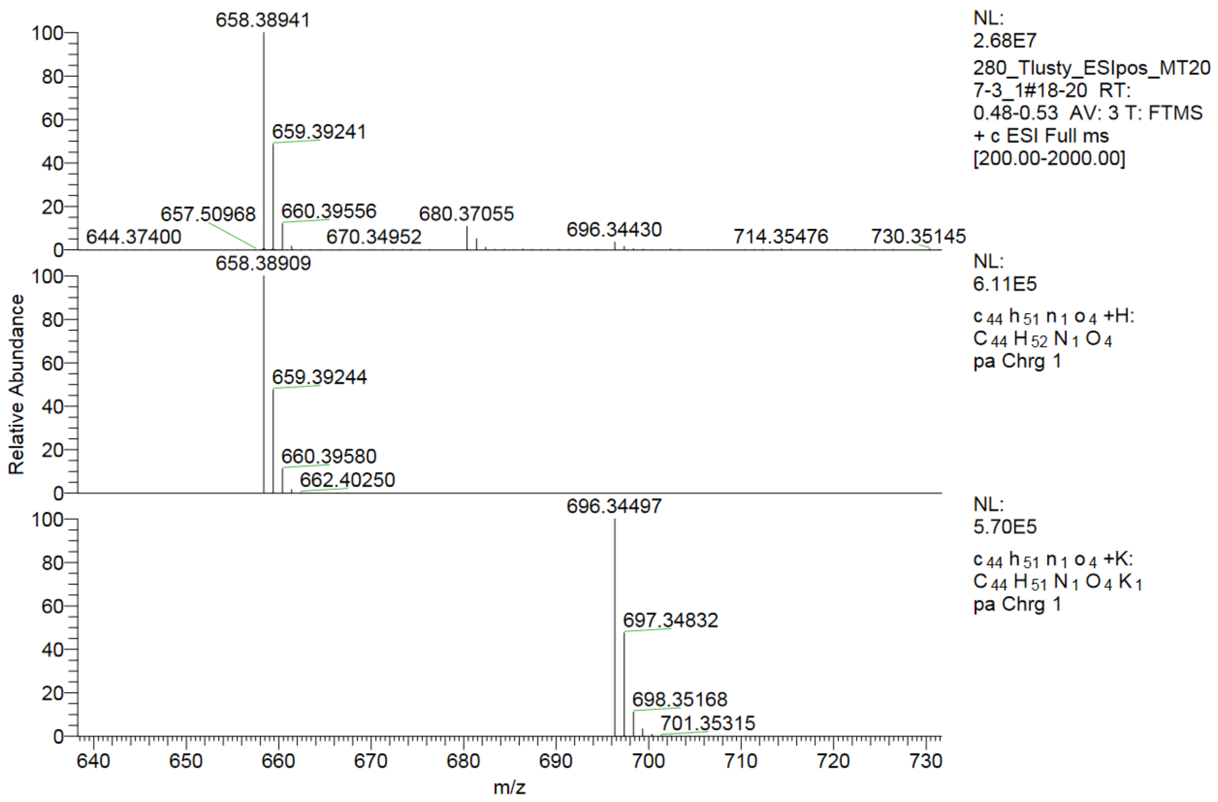

Figure S11: HRMS of compound **6a** (ESI+).

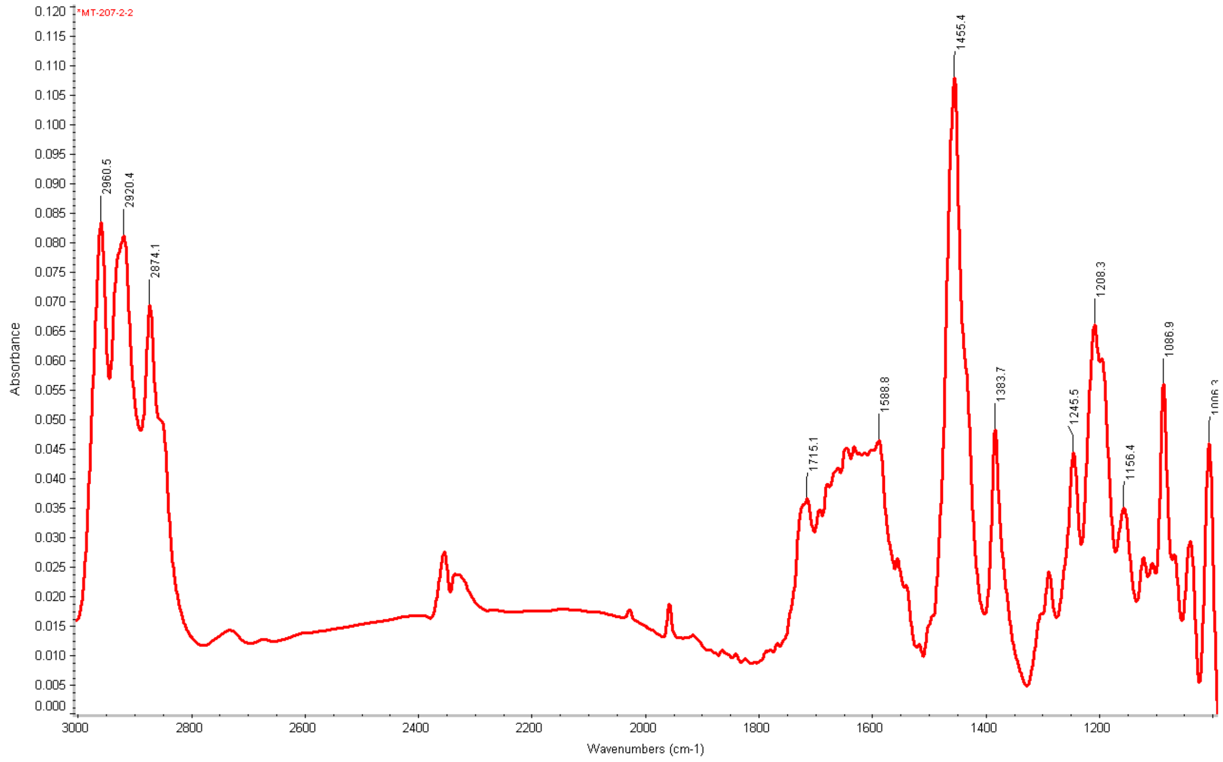

Figure S12: IR of compound **6a** (KBr).

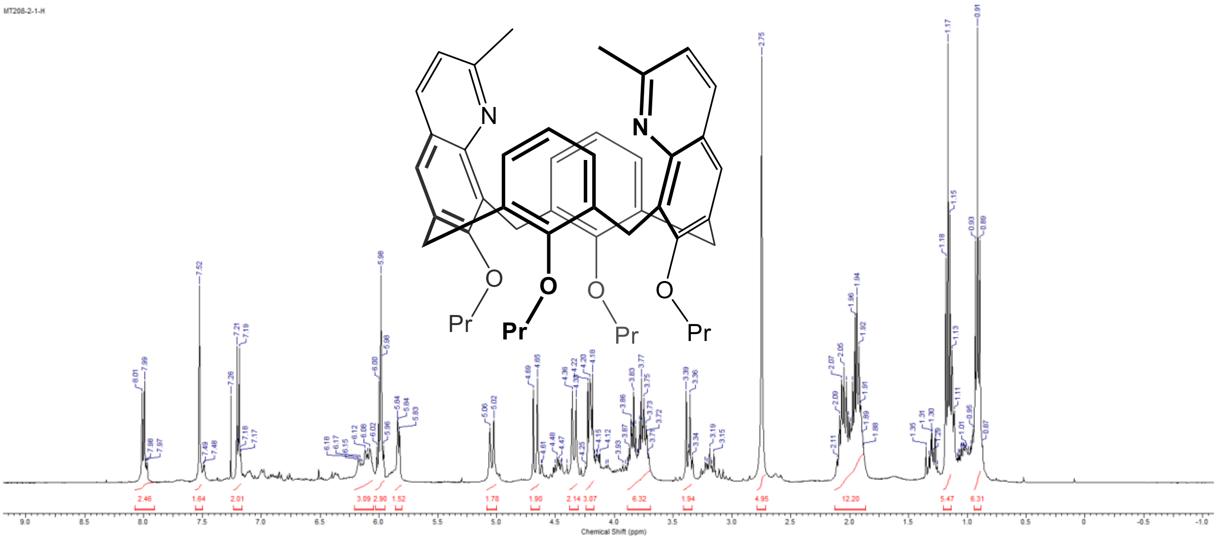

Figure S13: ^{1}H NMR of compound **14** ($CDCl_3$, 400 MHz).

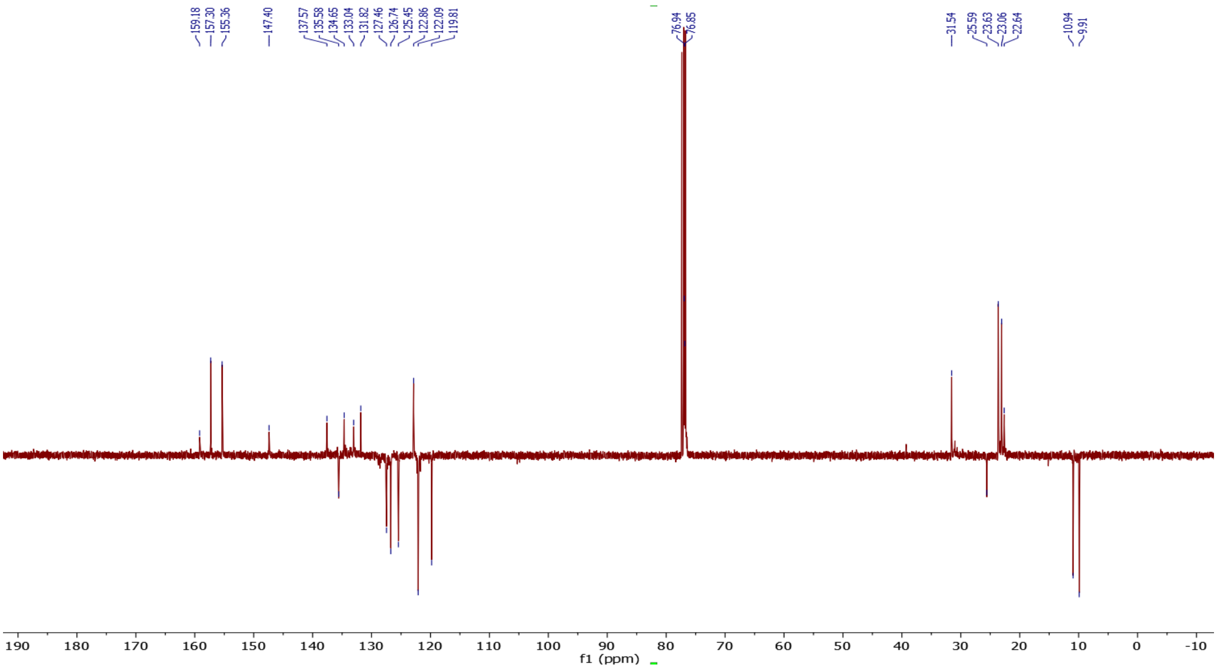

Figure S14: ^{13}C(APT) NMR of compound **14** ($CDCl_3$, 100 MHz).

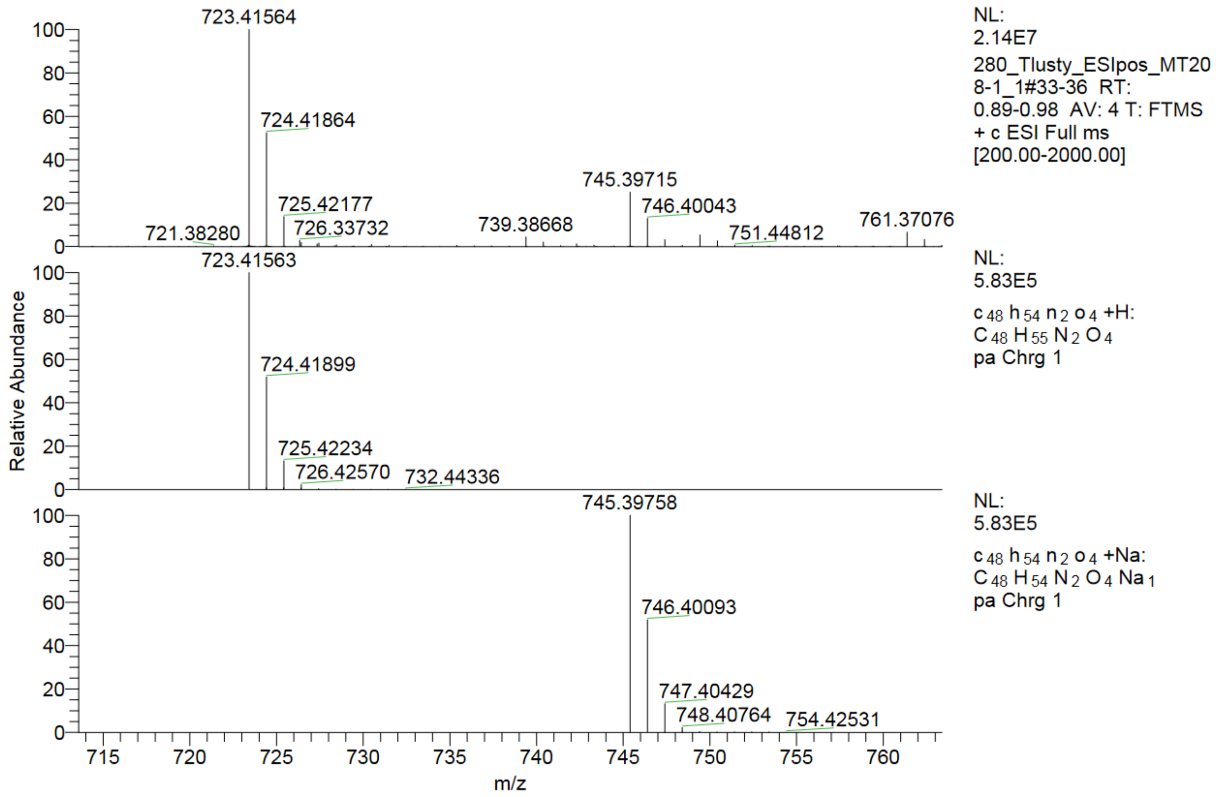

Figure S15: HRMS of compound **14** (ESI^{+}).

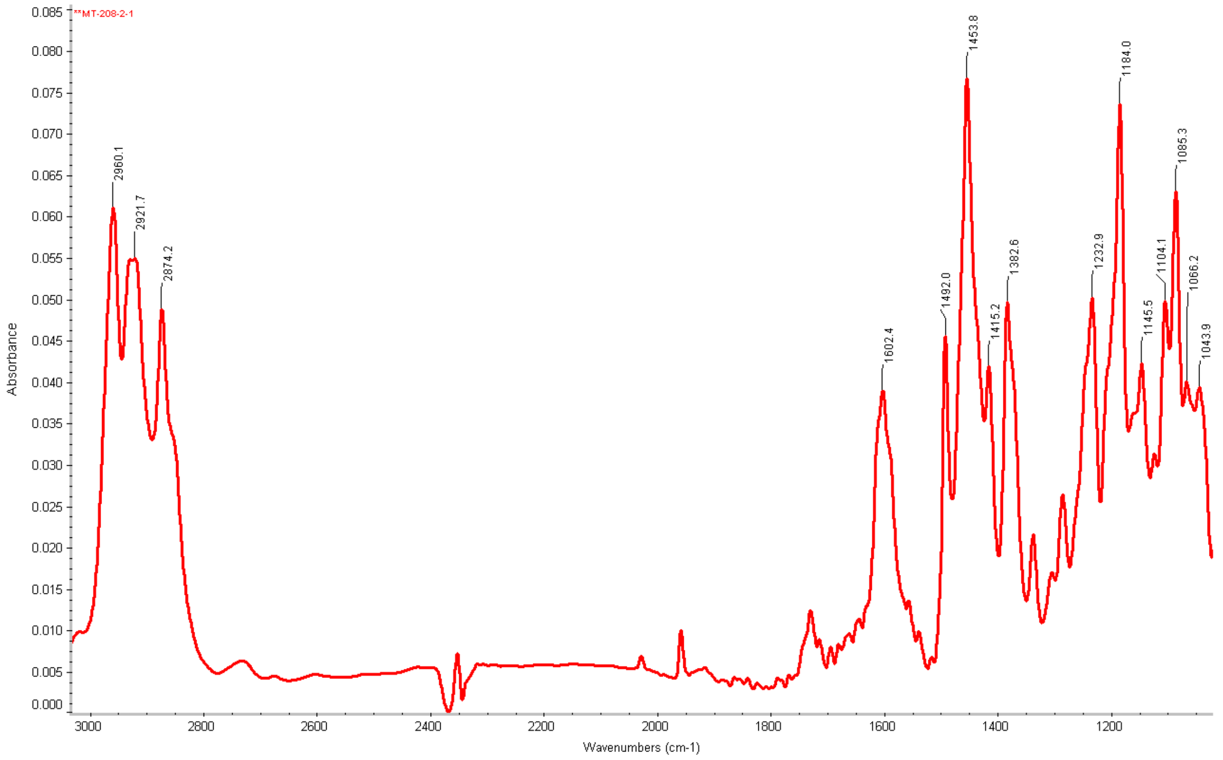

Figure S16: IR of compound **14** (KBr).

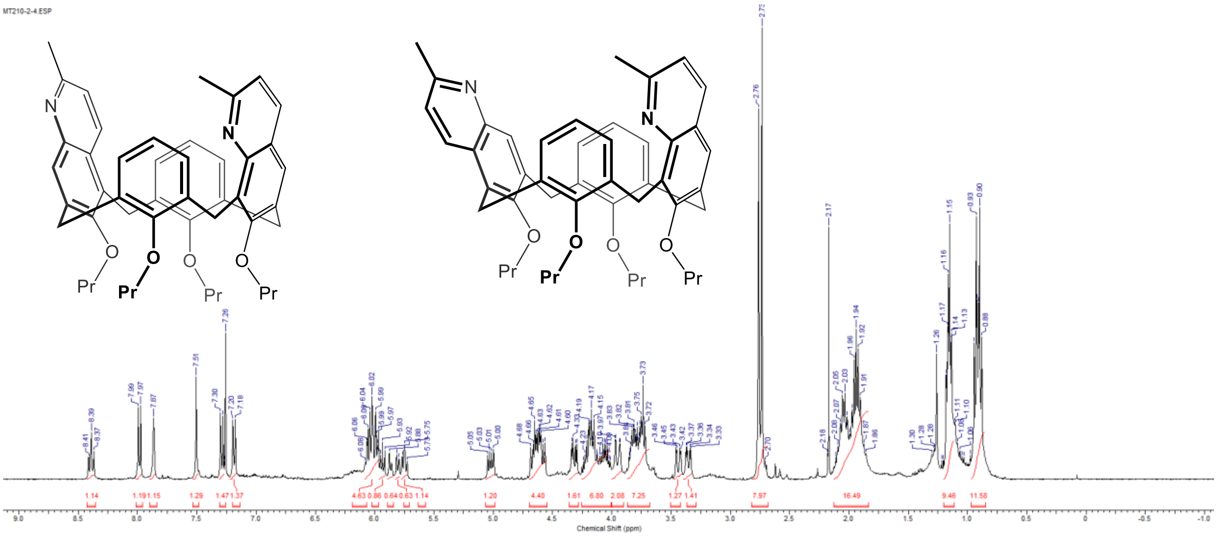

Figure S17: ^{1}H NMR of the mixture of compounds **15a** and **15b** ($CDCl_3$, 400 MHz).

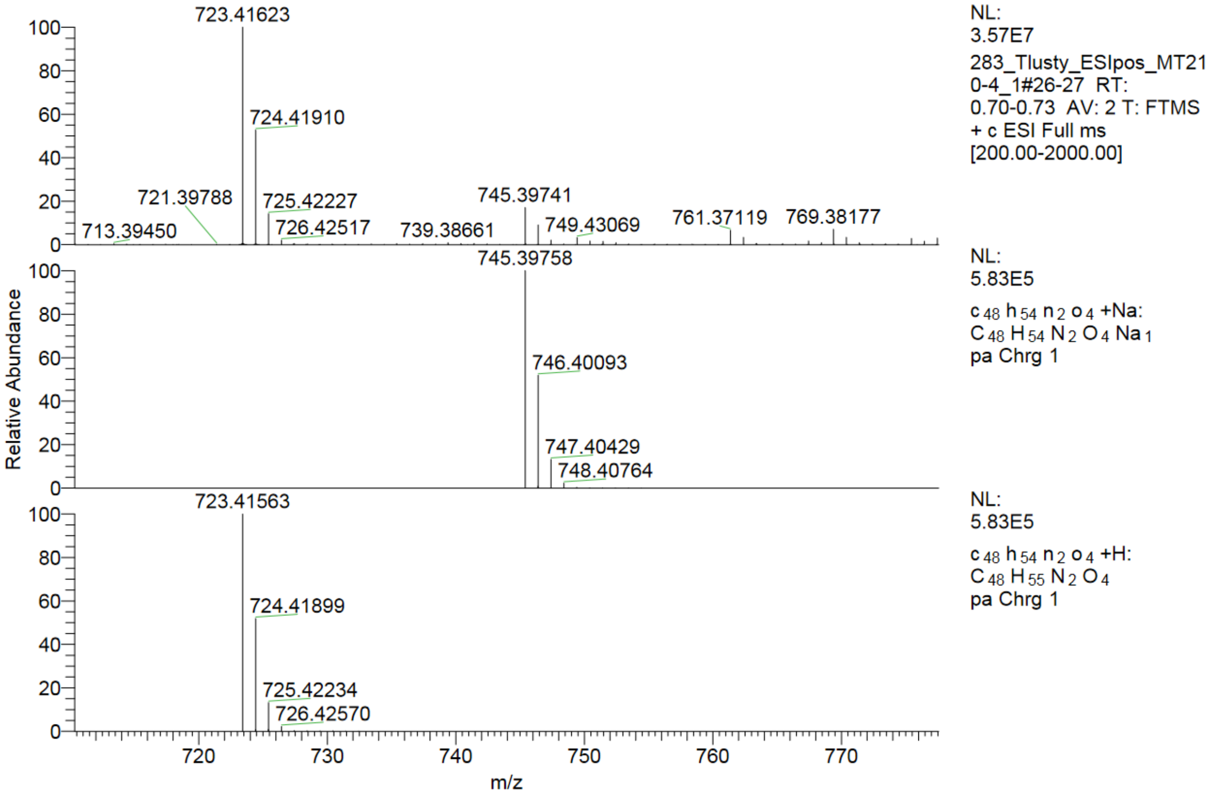

Figure S18: HRMS of the mixture of compounds **15a** and **15b** (ESI^{+}).

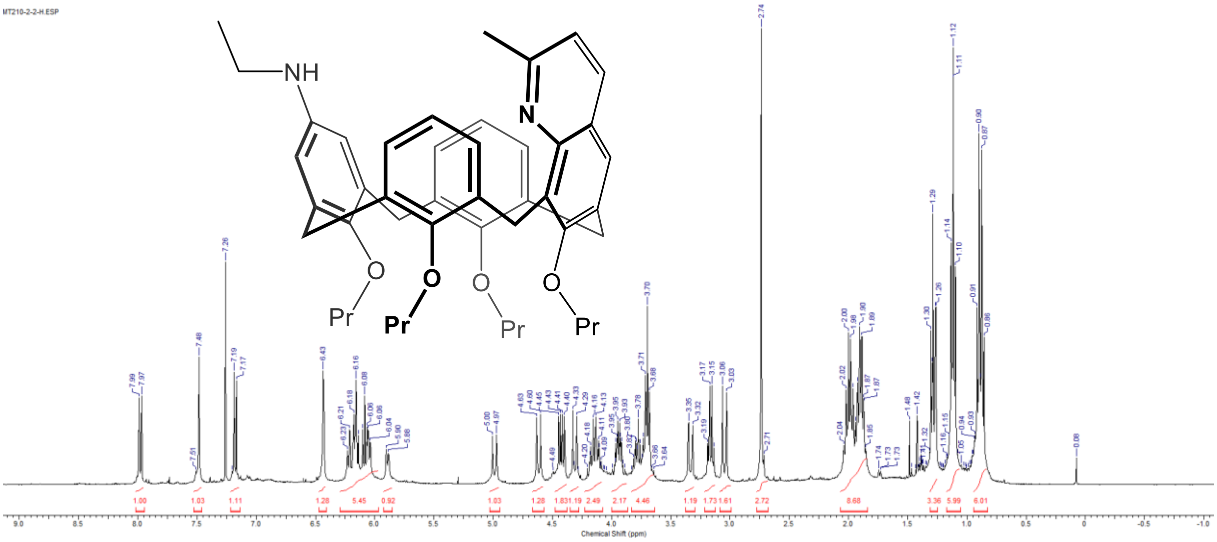

Figure S19: ^{1}H NMR of compound **15c** ($CDCl_3$, 400 MHz).

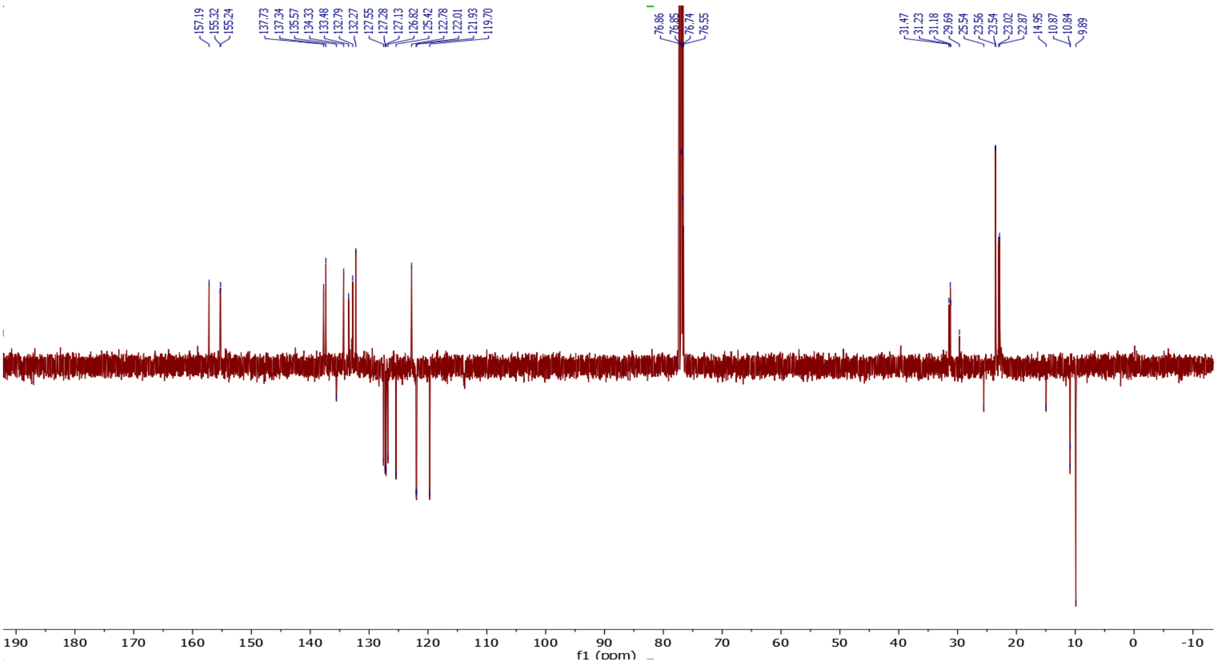

Figure S20: ^{13}C(APT) NMR of compound **15c** ($CDCl_3$, 100 MHz).

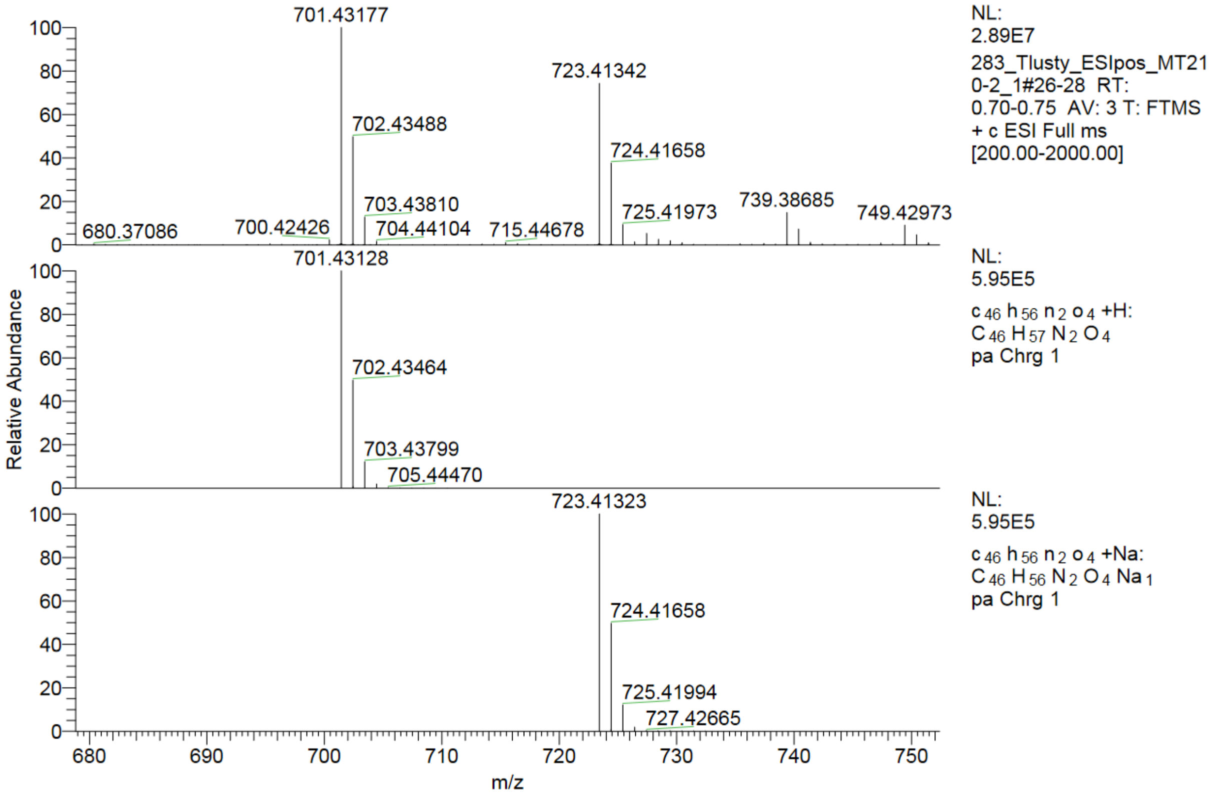

Figure S21: HRMS of compound **15c** (ESI+).

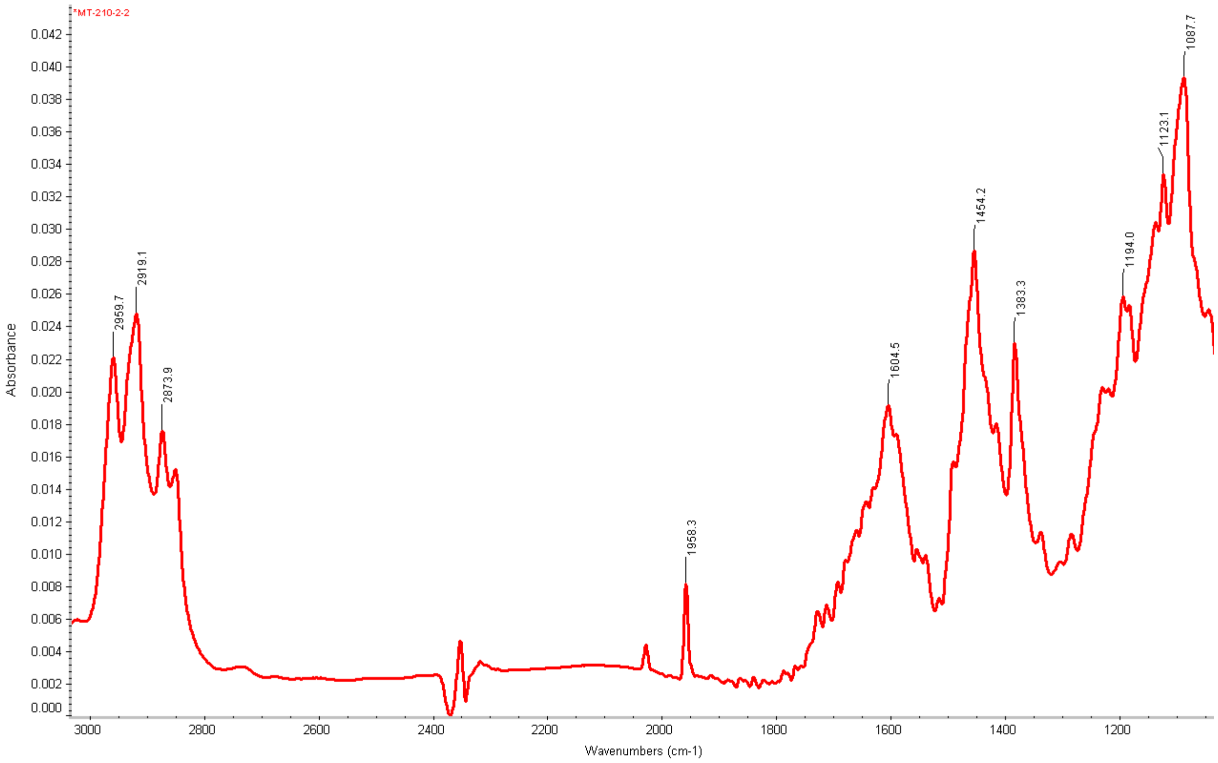

Figure S22: IR of compound **15c** (KBr).

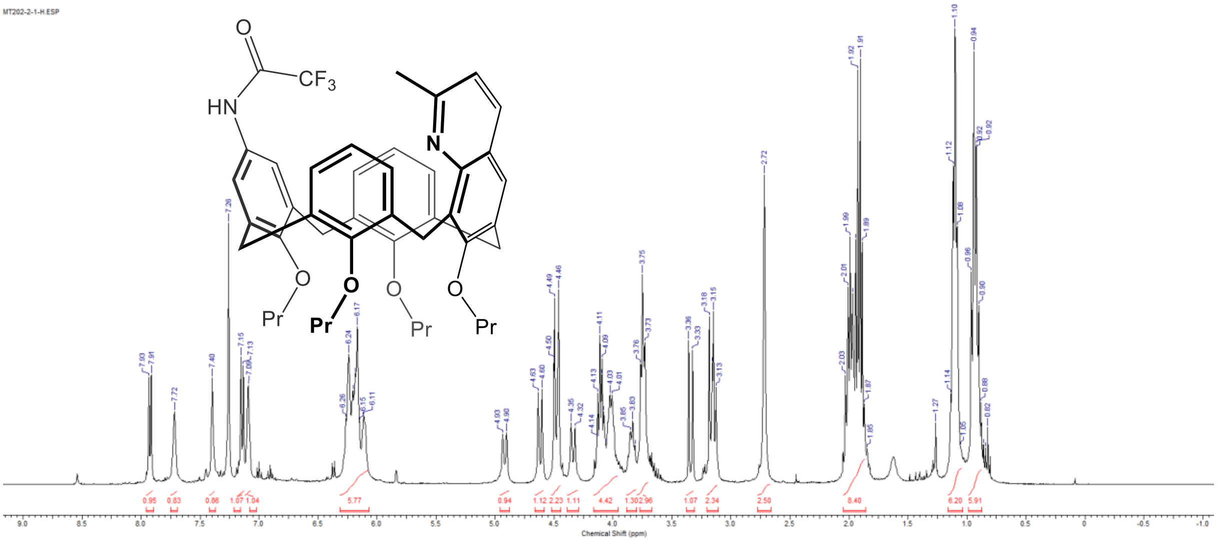

Figure S23: ^{1}H NMR of compound **16** ($CDCl_3$, 400 MHz).

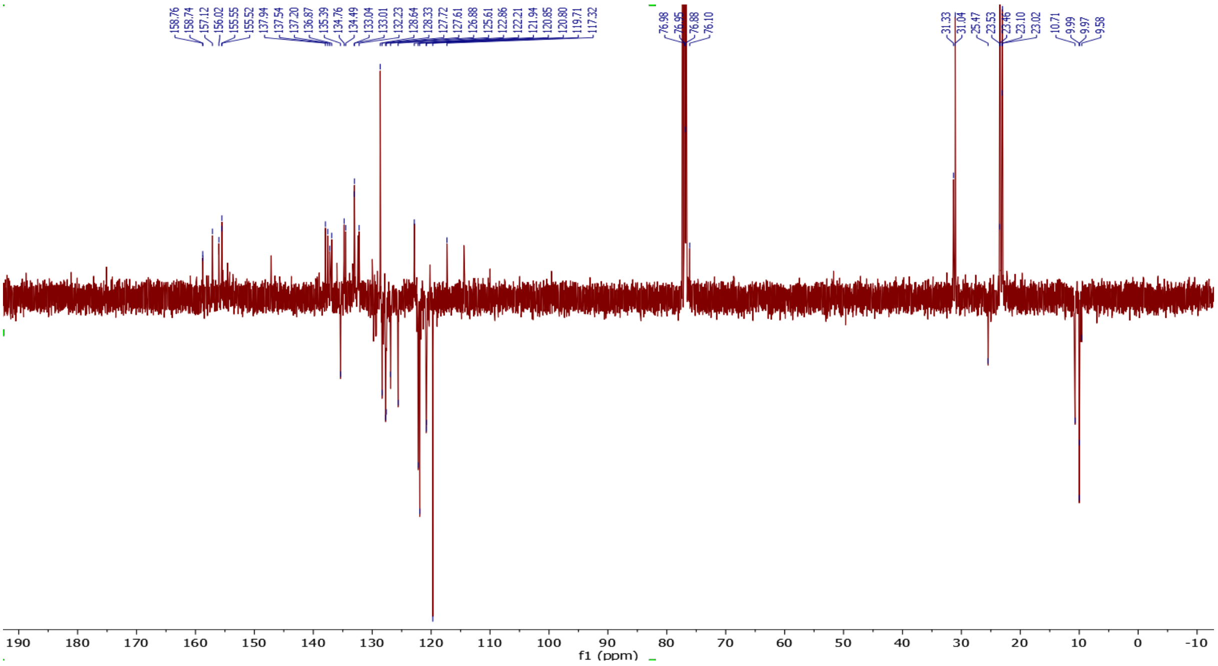

Figure S24: ^{13}C(APT) NMR of compound **16** ($CDCl_3$, 100 MHz).

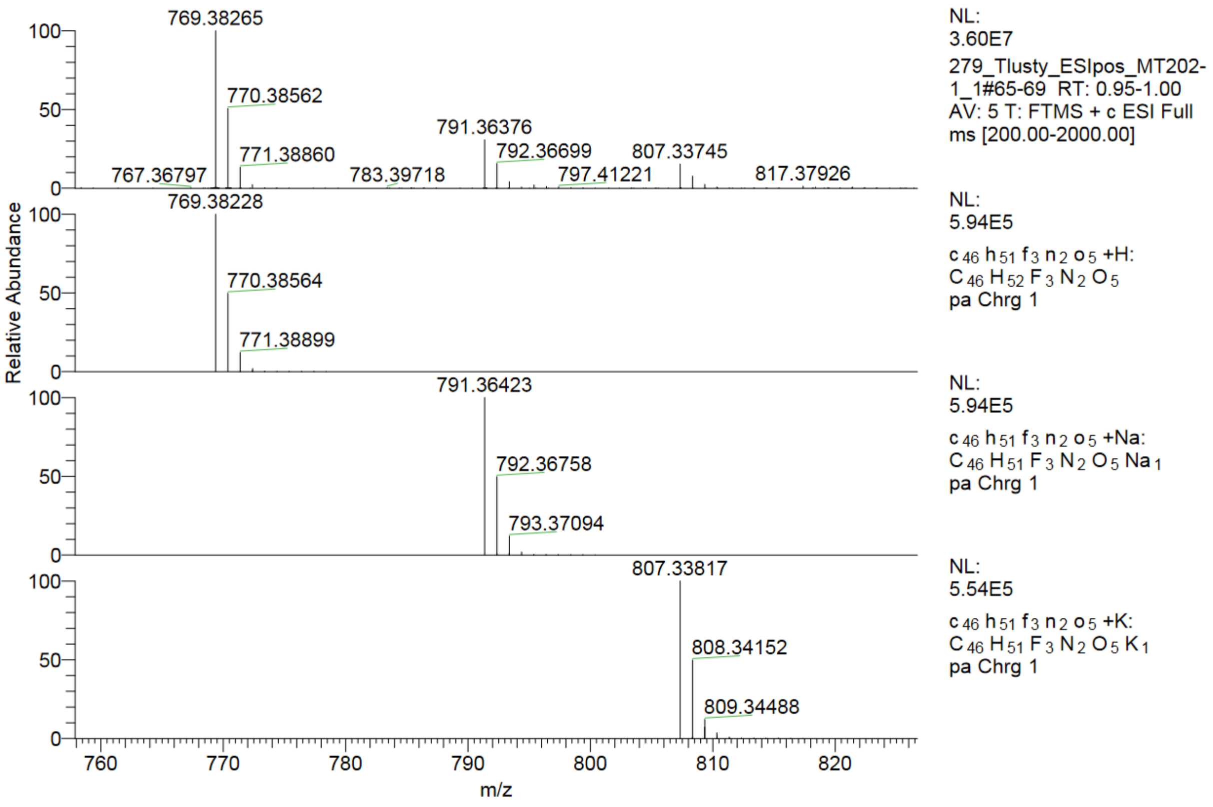

Figure S25: HRMS of compound **16** (ESI+).

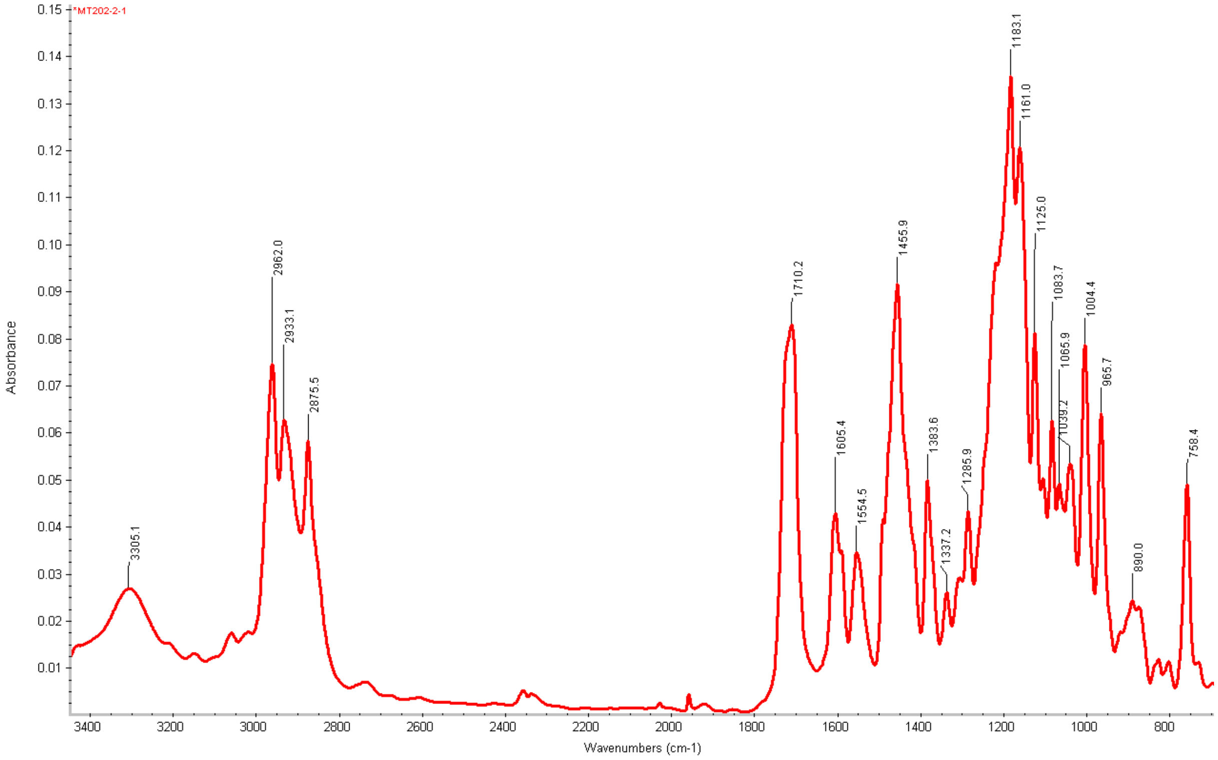

Figure S26: IR of compound **16** (KBr).

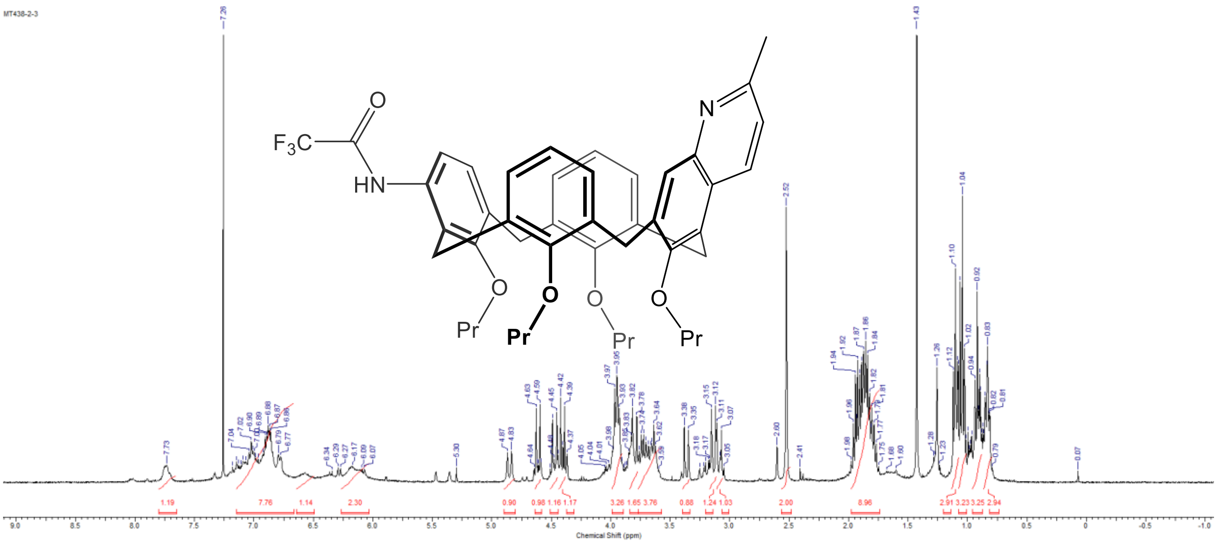

Figure S27: ^{1}H NMR of compound **17a** ($CDCl_3$, 400 MHz).

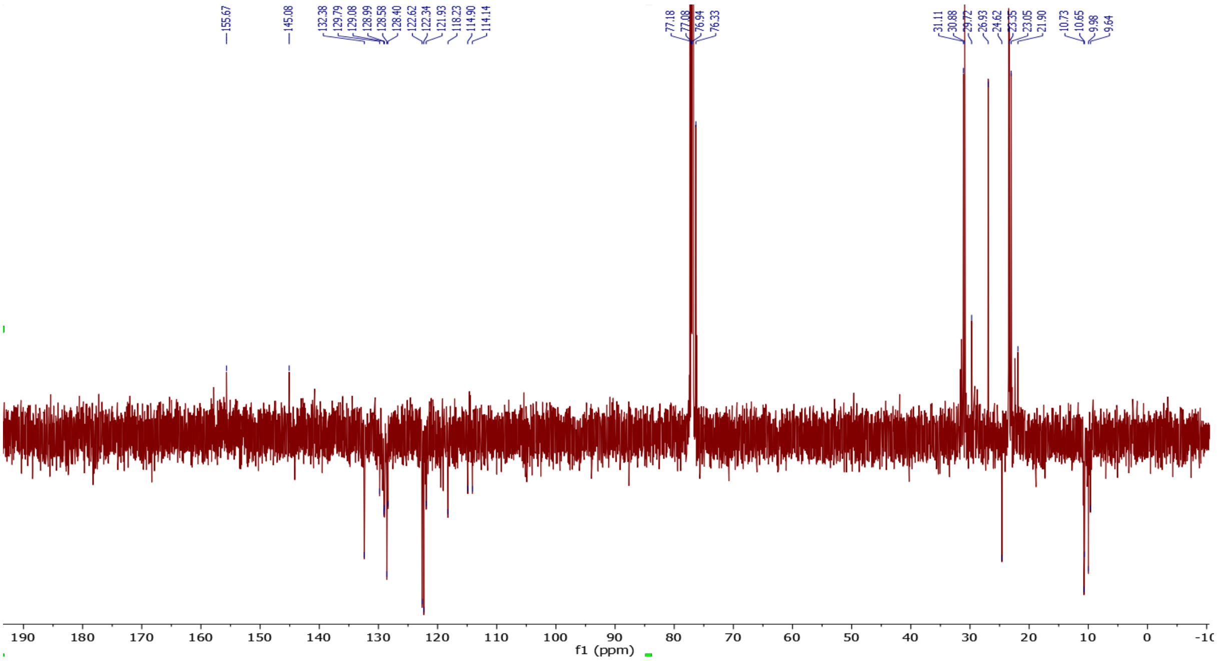

Figure S28: ^{13}C(APT) NMR of compound **17a** ($CDCl_3$, 100 MHz).

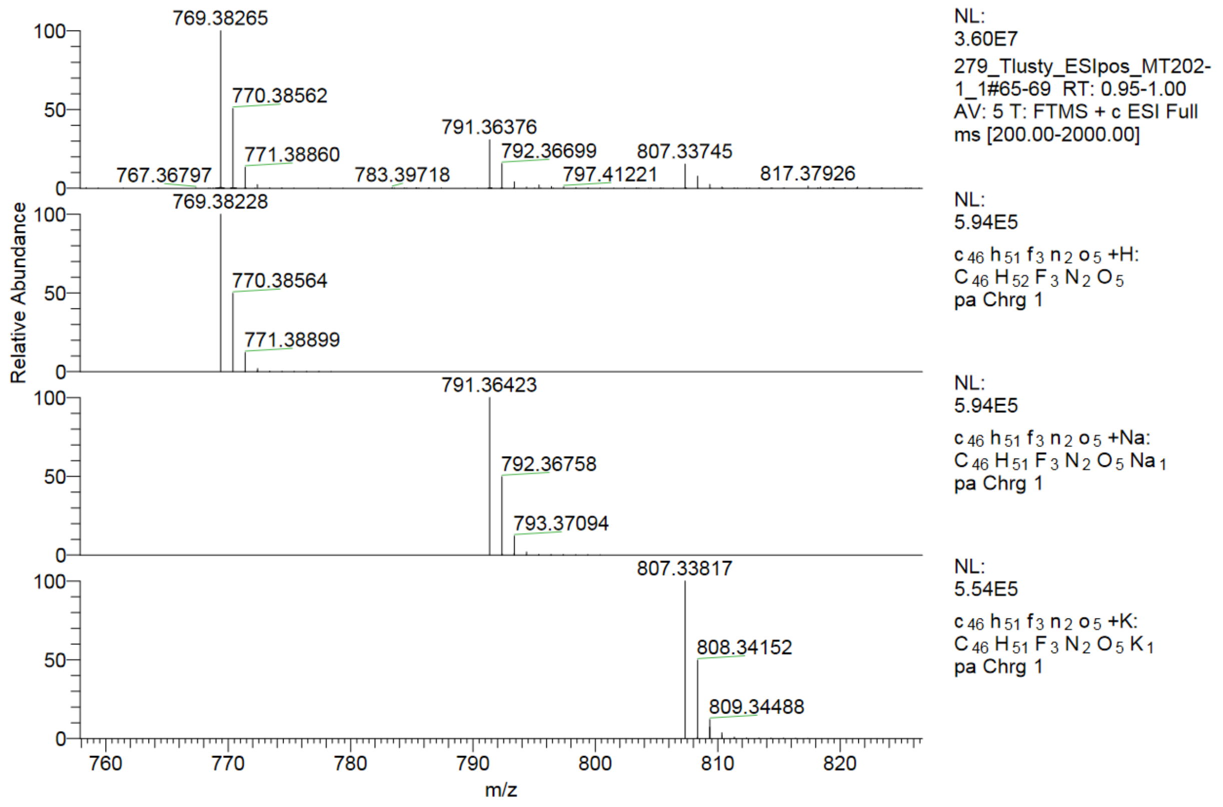

Figure S29: HRMS of compound **17a** (ESI+).

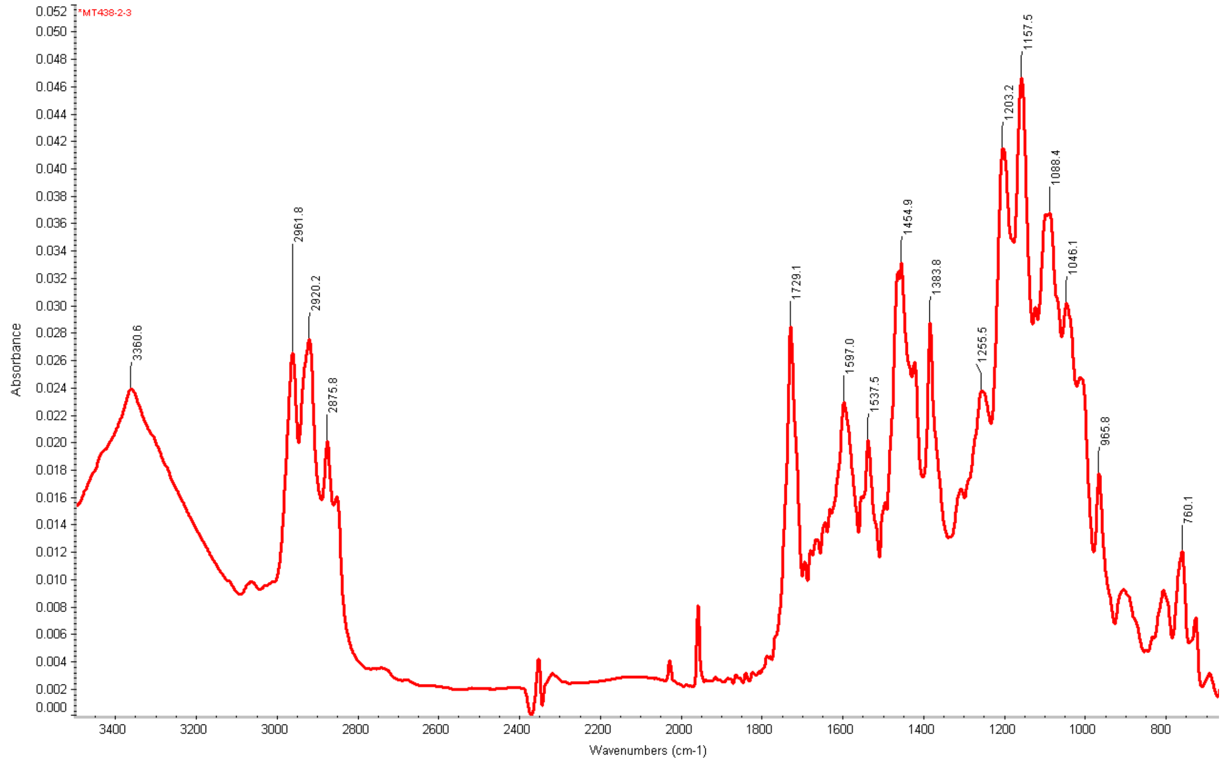

Figure S30: IR of compound **17a** (KBr).

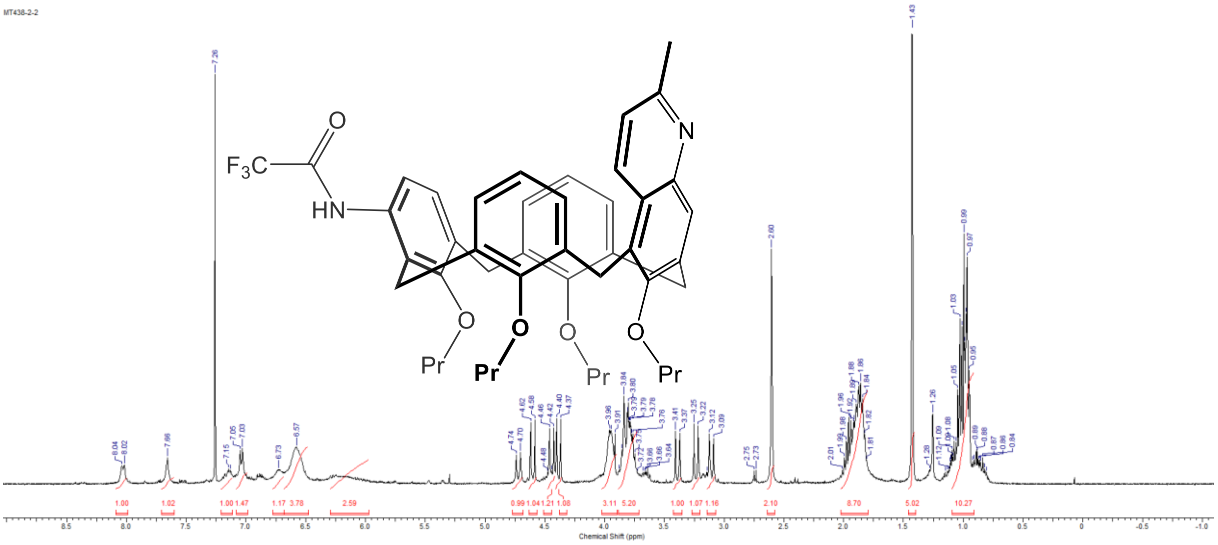

Figure S31: ^{1}H NMR of compound **17b** ($CDCl_3$, 400 MHz).

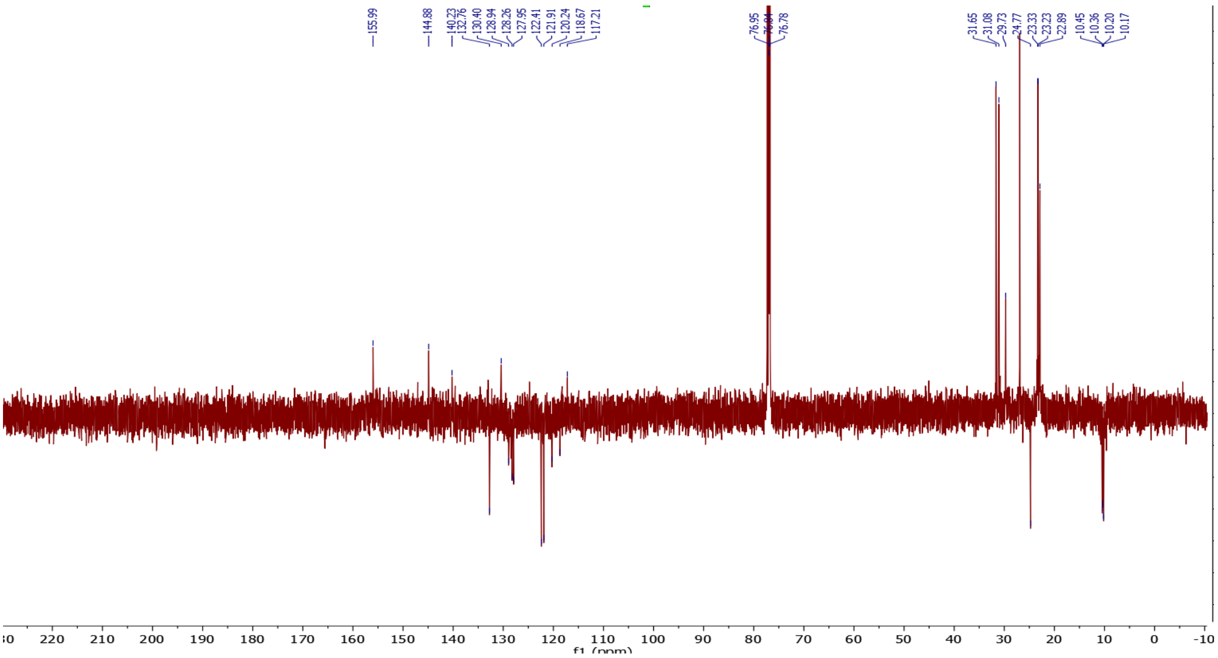

Figure S32: ^{13}C(APT) NMR of compound **17b** ($CDCl_3$, 100 MHz).

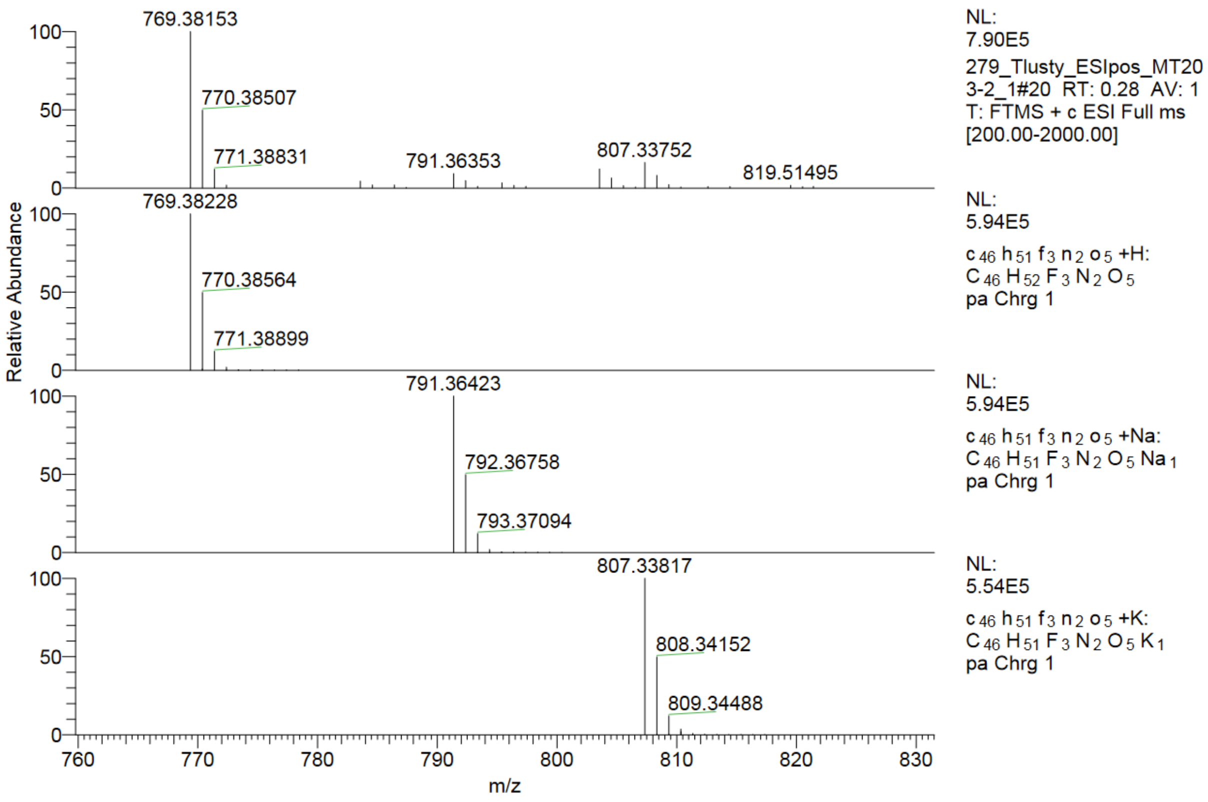

Figure S33: HRMS of compound **17b**(ESI^{+}).

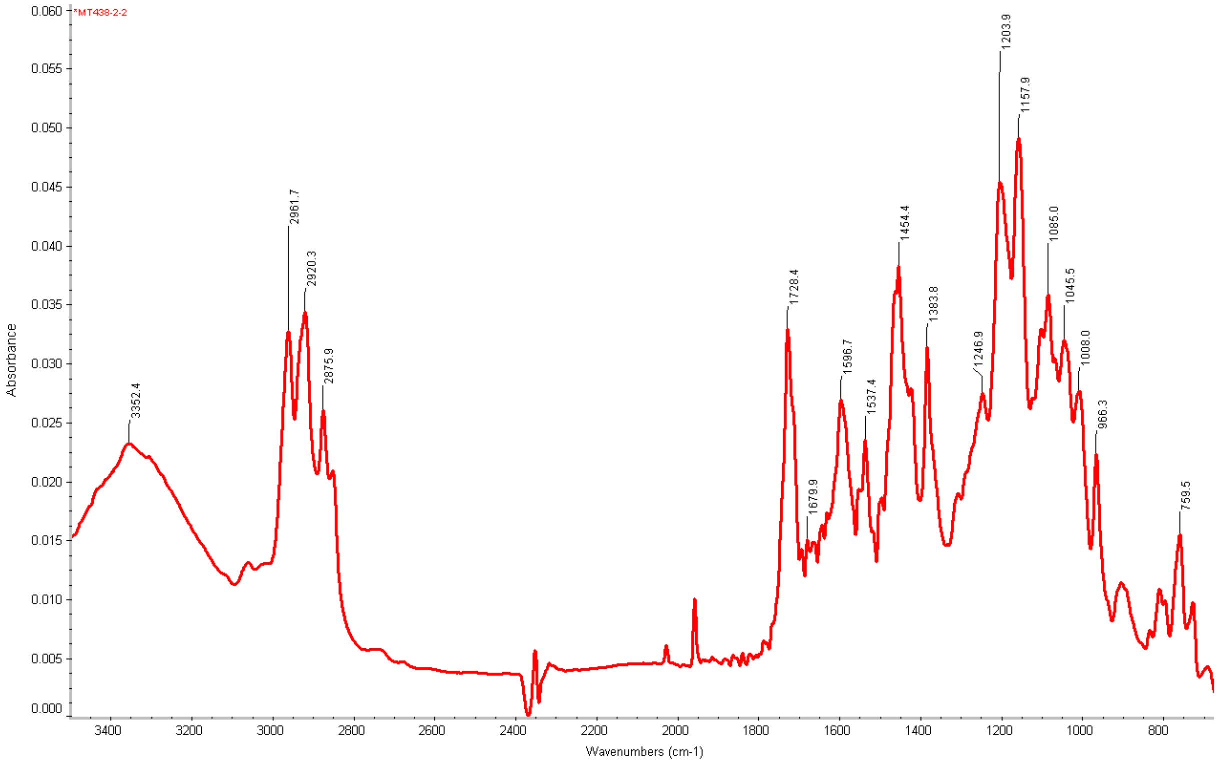

Figure S34: IR of compound **17b** (KBr).

2. Crystallographic data

*Crystallographic data for **16***

M = 800.94 g.mol^{-1}, triclinic system, space group P-1, a = 15.52150 (2) Å, b = 18.08924 (3) Å, c = 18.11720 (2) Å, α = 77.920 (3)°, β = 71.014 (2)°, γ = 64.684 (2)° Z = 4, V = 4333.46 (9) Å^3, D_c = 1.228 g.cm^{-3}, μ(Cu-Kα) = 0.73 mm^{-1}, crystal dimensions of 0.41 × 0.29 × 0.26 mm. Data were collected at 200 (2) K on a Bruker D8 Venture Photon CMOS diffractometer with Incoatec microfocus sealed tube Cu-$K\alpha$ radiation. The structure was solved by charge flipping methods [1] and anisotropically refined by full matrix least squares on F squared using the CRYSTALS [2] to final value R = 0.085 and wR = 0.242 using 15813 independent reflections (θ_{max} = 68.4°), 1370 parameters and 481 restraints. The hydrogen atoms bonded to carbon atoms were placed in calculated positions refined with a riding constrains, while hydrogen atoms bonded to oxygen and nitrogen were refined using soft restraints. The disordered functional groups positions were found in difference electron density maps and refined with restrained geometry. MCE [3] was used for visualization of electron density maps. The occupancy of disordered functional group was constrained to full. The structure was deposited into Cambridge Structural Database under number CCDC 2215763.

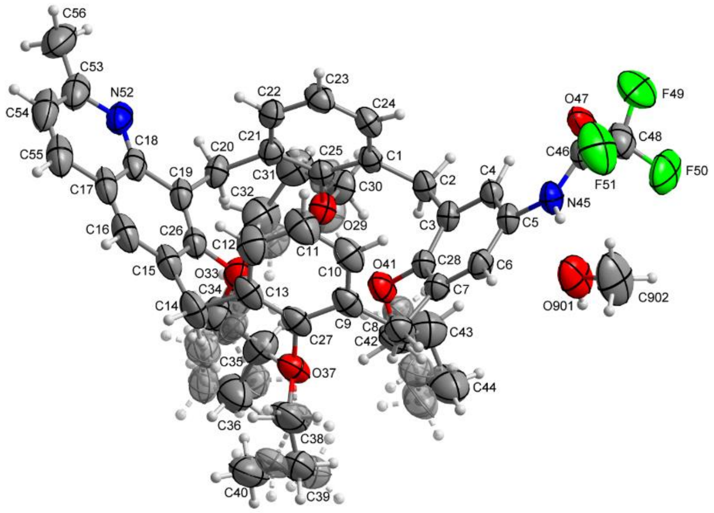

Figure S35: The molecule 1 of crystal structure of **16**, the ADPs drawn at 50% probability level. The weakly occupied atoms depicted as transparent. The atomic labels of weakly occupied atoms were omitted for clarity.

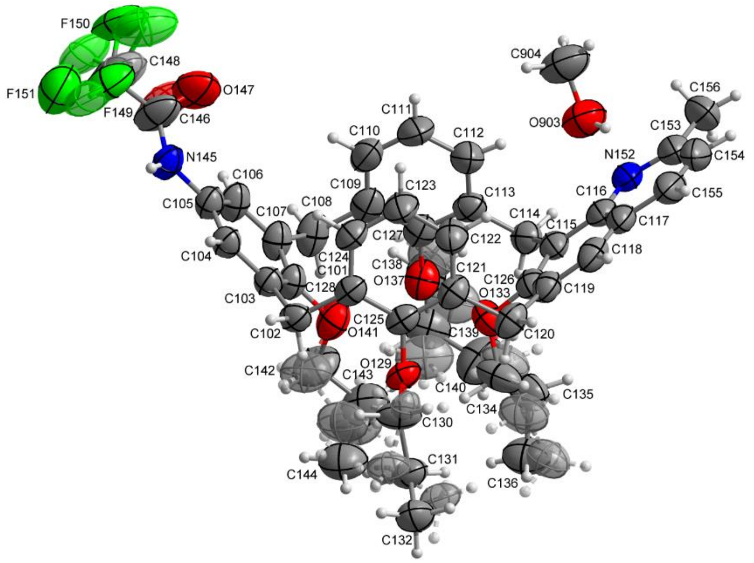

Figure S36: The molecule 2 of crystal structure of **16**, the ADPs drawn at 50% probability level. The weakly occupied atoms depicted as transparent. The atomic labels of weakly occupied atoms were omitted for clarity.

[1] Palatinus, L.; Chapuis, G., SUPERFLIP - a computer program for the solution of crystal structures by charge flipping in arbitrary dimensions. *Journal of Applied crystallography* **2007**, 40, 786-790.

[2] Betteridge, P. W.; Carruthers, J. R.; Cooper, R. I.; Prout, K.; Watkin, D. J., Software for guided crystal structure analysis. *Journal of Applied crystallography* **2003**, 36, 1487.

[3] Rohlicek, J.; Husak, M., *MCE2005* - a new version of a program for fast interactive visualization of electron and similar density maps optimized for small molecules. *Journal of Applied crystallography* **2007**, 40, 600-601.

3. Chiral separation

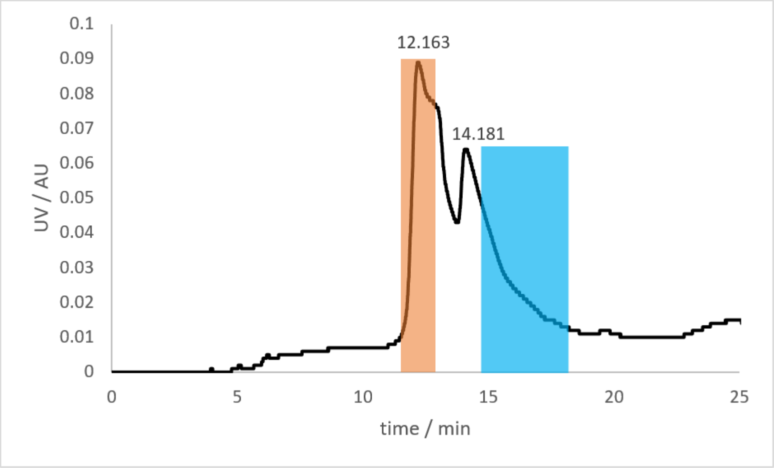

Figure S37: Preparative enantioseparation of **6a** on ChiralArt Amylose-SA (250 × 20 mm ID, 5 µm) in cyclohexane/DCM: 92/8, v/v at ambient temperature, flow rate 20 ml·min^{-1}, detection wavelength 350 nm, the collected fractions highlighted in orange (**6a_1**) and blue (**6a_2**).

4. Titration experiments

Calixarene **4a** was dissolved in a specified amount of $C_2D_2Cl_4$. 0.5 ml of calixarene solution was put in an NMR tube. To the calixarene solution (1.5 ml), a specific amount of *N*-methylpyridinium iodide (NMPI) was added. The aliquots of NMPI were gradually added to the NMR tube to achieve different calixarene/guest ratios (1:0.000-49.464), ensuring constant host concentration during the experiment. The complexation constant was determined by analyzing CIS of host protons using nonlinear curve-fitting procedure (program BindFit).

M(NMPI)	221.0415 g/mol	M(calix)	657.895 g/mol
m(NMPI)	0.02507 g	m(calix)	0.00163 g
c(NMPI)	0.07561 mol/l	c(calix)	0.00083 mol/l
V(C2D2Cl4	1.5 ml	V(C2D2Cl4	3 ml

	V (total) [ml]	V(additior	V(additior	c(NMPI) [r	c(calix) [m	c(calix)/c(N	shift [Hz]	shift 2 [Hz	shift 3 [Hz	shift 4 [Hz	shift 5 [Hz	shift 6 [Hz]
1	0.5000	0.0000	0.0000	0.00000	0.00083	0.00000	3430.01	2437.73	2268.31	1775.70	1755.74	1755.74
2	0.5012	0.0012	0.0012	0.00018	0.00083	0.21920	3422.57	2450.26	2267.14	1776.09	1754.57	1754.57
3	0.5024	0.0024	0.0012	0.00036	0.00083	0.43736	3418.27	2458.08	2264.79	1775.70	1753.00	1753.00
4	0.5036	0.0036	0.0012	0.00054	0.00083	0.65448	3415.14	2463.95	2264.01	1775.70	1751.83	1751.83
5	0.5056	0.0056	0.0020	0.00084	0.00083	1.01405	3412.40	2468.65	2264.40	1776.48	1752.22	1752.22
6	0.5096	0.0096	0.0040	0.00142	0.00083	1.72473	3408.10	2476.86	2262.83	1776.87	1750.66	1750.66
7	0.5176	0.0176	0.0080	0.00257	0.00083	3.11313	3404.18	2483.91	2262.44	1776.87	1749.87	1749.87
8	0.5276	0.0276	0.0100	0.00396	0.00083	4.78943	3400.66	2488.99	2262.44	1777.26	1749.09	1749.09
9	0.5476	0.0476	0.0200	0.00657	0.00083	7.95835	3396.36	2493.30	2262.05	1778.05	1749.87	1749.87
10	0.5776	0.0776	0.0300	0.01016	0.00083	12.30025	3392.84	2493.30	2262.83	1778.44	1750.66	1750.66
11	0.6176	0.1176	0.0400	0.01440	0.00083	17.43330	3388.92	2492.51	2263.23	1779.61	1751.44	1751.44
13	0.6976	0.1976	0.0800	0.02142	0.00083	25.93343	3383.84	2488.99	2265.96	1780.78	1752.61	1752.61
14	0.7876	0.2876	0.0900	0.02761	0.00083	33.43203	3379.92	2484.69	2267.53	1781.57	1754.57	1754.57
15	0.8876	0.3876	0.1000	0.03302	0.00083	39.98030	3376.40	2481.56	2269.09	1782.35	1755.74	1755.74
16	1.0876	0.5876	0.2000	0.04085	0.00083	49.46433	3371.32	2476.47	2271.44	1783.91	1757.31	1757.31

K11 12.99 M^{-1}
Error 0.234093 M^{-1}

K21 2798.4 M^{-1}
Error 38.86978 M^{-1}

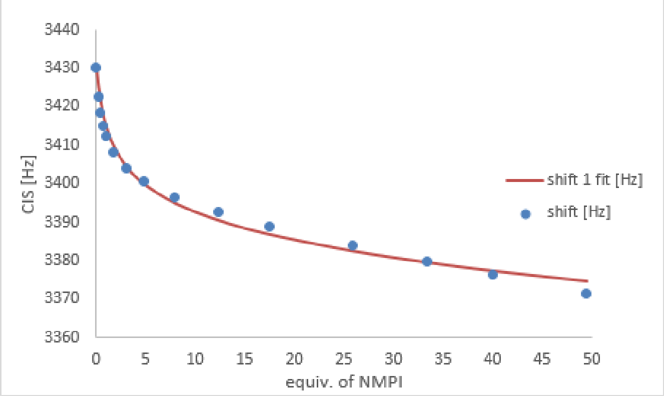

Figure S38:^{1}H NMR titration of compound **4a** with NMPI ($CDCl_3$, 400 MHz).

Calixarene **4a** was dissolved in a specified amount of $C_2D_2Cl_4$. 0.5 ml of calixarene solution was put in an NMR tube. To the calixarene solution (0.6 ml), a specific amount of *N*-methylquinolinium iodide (NMQI) was added. The aliquots of NMQI were gradually added to the NMR tube to achieve different calixarene/guest ratios (1:0.000-52.044), ensuring constant host concentration during the experiment. The complexation constant was determined by analyzing CIS of host protons using nonlinear curve-fitting procedure (program BindFit).

M(NMQI)	271.10147	g/mol	M(calix)	605.819	g/mol
m(NMQI)	0.01522	g	m(calix)	0.00119	g
c(NMQI)	0.09357	mol/l	c(calix)	0.00098	mol/l
V(C2D2Cl4_NMQI)	0.6	ml	V(C2D2Cl4_cali	2	ml

	V (total) [m	V(addition, total) [n	V(addition) [ml]	c(NMQI) [mol/l]	c(calix) [mol/l]	c(calix)/c(NM	shift [Hz]	shift 2 [Hz	shift 3 [Hz	shift 4 [Hz	shift 5 [Hz	shift 6 [Hz]
1	0.5000	0.0000	0.0000	0.00000	0.00098	0.00000	3276.30	3268.74	3183.39	3150.05	3017.76	2619.60
2	0.5020	0.0020	0.0020	0.00037	0.00098	0.37956	3276.09	3268.57	3182.53	3150.10	3018.54	2622.67
3	0.5040	0.0040	0.0020	0.00074	0.00098	0.75611	3275.87	3268.00	3181.63	3150.42	3019.41	2625.95
4	0.5060	0.0060	0.0020	0.00111	0.00098	1.12969	3276.64	3267.16	3180.64	3150.60	3020.38	2629.77
5	0.5140	0.0140	0.0080	0.00255	0.00098	2.59491	3277.17	3263.98	3176.98	3151.00	3023.66	2642.30
6	0.5220	0.0220	0.0080	0.00394	0.00098	4.01522	3278.24	3260.55	3174.06	3151.60	3027.30	2655.15
7	0.5320	0.0320	0.0100	0.00563	0.00098	5.73054	3279.63	3258.74	3170.15	3152.40	3031.06	2669.96
8	0.5520	0.0520	0.0200	0.00881	0.00098	8.97474	3280.84	3253.30	3161.43	3153.08	3037.97	2694.20
9	0.5720	0.0720	0.0200	0.01178	0.00098	11.99206	3282.23	3248.91	3157.72	3153.73	3043.76	2719.90
10	0.6020	0.1020	0.0300	0.01585	0.00098	16.14214	3283.52	3243.23	3150.16	3154.69	3050.94	2749.94
11	0.6320	0.1320	0.0300	0.01954	0.00098	19.89822	3285.17	3238.65	3144.38	3155.52	3057.27	2774.56
12	0.6820	0.1820	0.0500	0.02497	0.00098	25.42403	3286.87	3232.35	3138.37	3156.45	3065.15	2807.91
13	0.7520	0.2520	0.0700	0.03136	0.00098	31.92568	3288.84	3225.66	3127.62	3157.19	3073.41	2844.94
14	0.8420	0.3420	0.0900	0.03801	0.00098	38.69648	3291.23	3219.22	3119.91	3158.00	3080.36	2877.85
15	0.9420	0.4420	0.1000	0.04390	0.00098	44.70219	3291.53	3214.54	3114.54	3158.34	3085.54	2902.13
16	1.0420	0.5420	0.1000	0.04867	0.00098	49.55518	3293.17	3211.75	3110.35	3158.78	3089.61	2921.88
17	1.1020	0.6020	0.0600	0.05111	0.00098	52.04420	3293.1	3209.12	3108.11	3159.06	3091.71	2928.27

K	12.24 M^{-1}
Error	0.04350096 M^{-1}

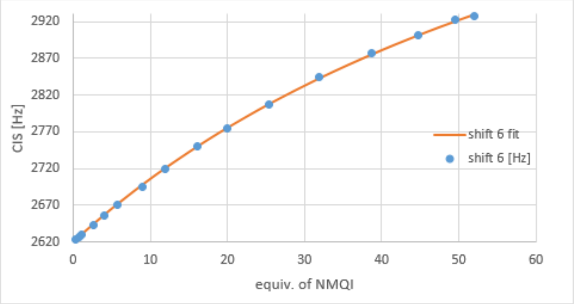

Figure S39:^{1}H NMR titration of compound **4a** with NMQI ($C_2D_2Cl_4$, 400 MHz).

Calixarene **4a** was dissolved in a specified amount of $C_2D_2Cl_4$. 0.5 ml of calixarene solution was put in an NMR tube. To the calixarene solution (0.6 ml), a specific amount of *N*-methylisoquinolinium iodide (NMII) was added. The aliquots of NMII were gradually added to the NMR tube to achieve different calixarene/guest ratios (1:0.000-50.121), ensuring constant host concentration during the experiment. The complexation constant was determined by analyzing CIS of host protons using nonlinear curve-fitting procedure (program BindFit).

M(NMII)	271.10147 g/mol	M(calix)	657.895 g/mol
m(NMII)	0.01690 g	m(calix)	0.00149 g
c(NMII)	0.10390 mol/l	c(calix)	0.00113 mol/l
V(C2D2Cl4_NMII)	0.6 ml	V(C2D2Cl4_cali	2 ml

	V (total) [m	V(addition, total) [n	V(addition) [ml]	c(NMII) [mol/l]	c(calix) [mol/l]	c(calix)/c(NM	shift [Hz]	shift 2 [Hz
1	0.5000	0.0000	0.0000	0.00000	0.00113	0.00000	3707.03	3390.49
2	0.5020	0.0020	0.0020	0.00041	0.00113	0.36554	3705.07	3388.92
3	0.5040	0.0040	0.0020	0.00082	0.00113	0.72817	3705.07	3388.53
4	0.5060	0.0060	0.0020	0.00123	0.00113	1.08794	3704.29	3388.14
5	0.5140	0.0140	0.0080	0.00283	0.00113	2.49901	3701.94	3386.18
6	0.5220	0.0220	0.0080	0.00438	0.00113	3.86684	3700.77	3385.01
7	0.5320	0.0320	0.0100	0.00625	0.00113	5.51877	3699.60	3383.45
8	0.5520	0.0520	0.0200	0.00979	0.00113	8.64307	3696.86	3381.10
9	0.5720	0.0720	0.0200	0.01308	0.00113	11.54889	3694.12	3378.75
10	0.6020	0.1020	0.0300	0.01760	0.00113	15.54560	3690.99	3376.01
11	0.6320	0.1320	0.0300	0.02170	0.00113	19.16288	3688.25	3373.66
12	0.6820	0.1820	0.0500	0.02773	0.00113	24.48448	3685.15	3370.14
13	0.7520	0.2520	0.0700	0.03482	0.00113	30.74586	3680.81	3366.62
14	0.8420	0.3420	0.0900	0.04220	0.00113	37.26644	3677.68	3363.88
15	0.9420	0.4420	0.1000	0.04875	0.00113	43.05021	3674.55	3360.75
16	1.0420	0.5420	0.1000	0.05404	0.00113	47.72385	3672.60	3358.80
17	1.1020	0.6020	0.0600	0.05676	0.00113	50.12089	3671.03	3357.23

K	16.59 M^{-1}
Error	0.44220645 M^{-1}

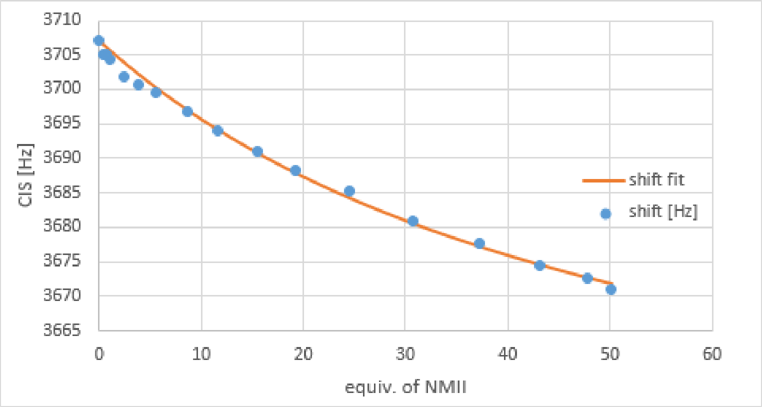

Figure S40:^{1}H NMR titration of compound **4a** with NMII ($C_2D_2Cl_4$, 400 MHz).

Calixarene **6a_1** was dissolved in a specified amount of $C_2D_2Cl_4$. 0.5 ml of calixarene solution was put in an NMR tube. To the calixarene solution (0.6 ml), a specific amount of (*S*)-*N*-methylnicotinium iodide (NMNI) was added. The aliquots of NMII were gradually added to the NMR tube to achieve different calixarene/guest ratios (1:0.000-51.573), ensuring constant host concentration during the experiment. The complexation constant was determined by analyzing CIS of host protons using nonlinear curve-fitting procedure (program BindFit).

M(NMNI)	304.17547	g/mol	M(calix)	657.895	g/mol
m(NMNI)	0.01896	g	m(calix)	0.00133	g
c(NMNI)	0.10389	mol/l	c(calix)	0.00101	mol/l
V(C2D2Cl4_NMNI)	0.6	ml	V(C2D2Cl4_cali	2	ml

	V (total) [m	V(addition, total) [r	V(addition) [ml]	c(NMNI) [mol/l]	c(calix) [mol/l]	c(calix)/c(NM	shift [Hz]
1	0.5000	0.0000	0.0000	0.00000	0.00101	0.00000	3422.73
2	0.5006	0.0006	0.0006	0.00012	0.00101	0.12319	3422.22
3	0.5012	0.0012	0.0006	0.00025	0.00101	0.24608	3421.30
4	0.5020	0.0020	0.0008	0.00041	0.00101	0.40947	3421.13
5	0.5028	0.0028	0.0008	0.00058	0.00101	0.57235	3420.91
6	0.5042	0.0042	0.0014	0.00087	0.00101	0.85614	3420.91
7	0.5056	0.0056	0.0014	0.00115	0.00101	1.13836	3420.13
8	0.5136	0.0136	0.0080	0.00275	0.00101	2.72152	3418.90
9	0.5236	0.0236	0.0100	0.00468	0.00101	4.63244	3417.88
10	0.5536	0.0536	0.0300	0.01006	0.00101	9.95100	3413.15
11	0.5936	0.0936	0.0400	0.01638	0.00101	16.20615	3409.22
12	0.6436	0.1436	0.0500	0.02318	0.00101	22.93170	3406.23
13	0.7136	0.2136	0.0700	0.03110	0.00101	30.76410	3403.47
14	0.8036	0.3036	0.0900	0.03925	0.00101	38.82931	3401.27
15	0.9036	0.4036	0.1000	0.04640	0.00101	45.90635	3399.54
16	1.0036	0.5036	0.1000	0.05213	0.00101	51.57306	3398.51

K	37.26	M^{-1}
Error	2.3943276	M^{-1}

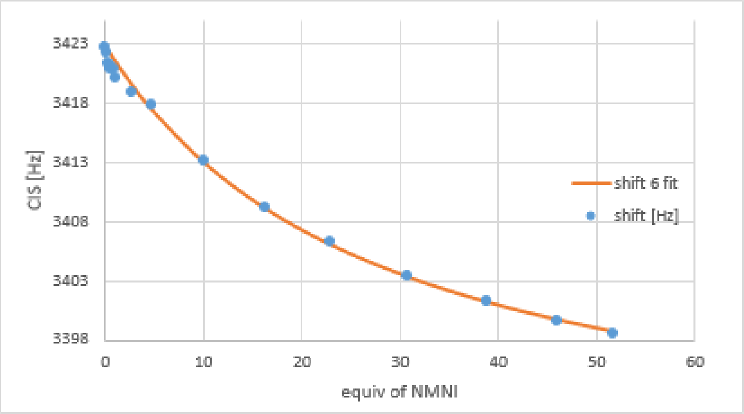

Figure S41:^{1}H NMR titration of compound **6a_1** with NMNI ($C_2D_2Cl_4$, 400 MHz).

Calixarene **6a_2** was dissolved in a specified amount of $C_2D_2Cl_4$. 0.5 ml of calixarene solution was put in an NMR tube. To the calixarene solution (0.6 ml), a specific amount of (*S*)-*N*-methylnicotinium iodide (NMNI) was added. The aliquots of NMII were gradually added to the NMR tube to achieve different calixarene/guest ratios (1:0.000-50.221), ensuring constant host concentration during the experiment. The complexation constant was determined by analyzing CIS of host protons using nonlinear curve-fitting procedure (program BindFit).

M(NMNI)	304.17547	g/mol	M(calix)	657.895	g/mol
m(NMNI)	0.01999	g	m(calix)	0.00144	g
c(NMNI)	0.10953	mol/l	c(calix)	0.00109	mol/l
V(C2D2Cl4_NMNI)	0.6	ml	V(C2D2Cl4_cali	2	ml

	V (total) [m	V(addition, total) [r	V(addition) [ml]	c(NMNI) [mol/l]	c(calix) [mol/l]	c(calix)/c(NM	shift [Hz]
1	0.5000	0.0000	0.0000	0.00000	0.00109	0.00000	3446.37
2	0.5006	0.0006	0.0006	0.00013	0.00109	0.11996	3445.88
3	0.5012	0.0012	0.0006	0.00026	0.00109	0.23962	3444.74
4	0.5020	0.0020	0.0008	0.00044	0.00109	0.39874	3443.76
5	0.5028	0.0028	0.0008	0.00061	0.00109	0.55735	3444.29
6	0.5042	0.0042	0.0014	0.00091	0.00109	0.83370	3443.76
7	0.5056	0.0056	0.0014	0.00121	0.00109	1.10852	3442.82
8	0.5136	0.0136	0.0080	0.00290	0.00109	2.65018	3436.86
9	0.5236	0.0236	0.0100	0.00494	0.00109	4.51101	3430.45
10	0.5536	0.0536	0.0300	0.01060	0.00109	9.69014	3421.52
11	0.5936	0.0936	0.0400	0.01727	0.00109	15.78132	3415.10
12	0.6436	0.1436	0.0500	0.02444	0.00109	22.33057	3410.25
13	0.7136	0.2136	0.0700	0.03279	0.00109	29.95766	3406.33
14	0.8036	0.3036	0.0900	0.04138	0.00109	37.81145	3403.36
15	0.9036	0.4036	0.1000	0.04892	0.00109	44.70297	3400.85
16	1.0036	0.5036	0.1000	0.05496	0.00109	50.22113	3399.44

K	72.09	M^{1}
Error	2.97126144	M^{1}

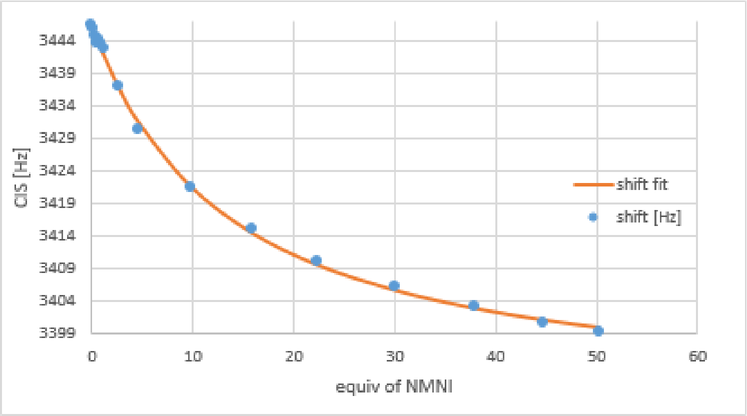

Figure S42:^{1}H NMR titration of compound **6a_2** with NMNI ($C_2D_2Cl_4$, 400 MHz).

Calixarene **16** was dissolved in a specified amount of $CDCl_3$. 0.5 ml of calixarene solution was put in an NMR tube. To the calixarene solution (0.6 ml), a specific amount of tetrabutylammonium benzoate (TBA-benzoate) was added. The aliquots of TBA-benzoate were gradually added to the NMR tube to achieve different calixarene/guest ratios (1:0.000-49.811), ensuring constant host concentration during the experiment. The complexation constant was determined by analyzing CIS of host protons using nonlinear curve-fitting procedure (program BindFit).

M(TBA-benzoate)	363.58	g/mol	M(calix)	768.918	g/mol
m(TBA-benzoate)	0.02544	g	m(calix)	0.00271	g
c(TBA-benzoate)	0.11662	mol/l	c(calix)	0.00117	mol/l
V(CDCl3_TBA-benzo	0.6	ml	V(CDCl3_calix)	3	ml

	V (total) [m	V(addition, total) [r	V(addition) [ml]	c(TBA-benzoate)	c(calix) [mol/l]	c(calix)/c(TBA	shift [Hz]
1	0.5000	0.0000	0.0000	0.00000	0.00117	0.00000	2825.01
2	0.5006	0.0006	0.0006	0.00014	0.00117	0.11898	2825.96
3	0.5012	0.0012	0.0006	0.00028	0.00117	0.23767	2827.33
4	0.5020	0.0020	0.0008	0.00046	0.00117	0.39548	2830.75
5	0.5028	0.0028	0.0008	0.00065	0.00117	0.55279	2831.14
6	0.5042	0.0042	0.0014	0.00097	0.00117	0.82688	2832.12
7	0.5056	0.0056	0.0014	0.00129	0.00117	1.09946	2833.68
8	0.5136	0.0136	0.0080	0.00309	0.00117	2.62852	2841.21
9	0.5236	0.0236	0.0100	0.00526	0.00117	4.47415	2852.36
10	0.5536	0.0536	0.0300	0.01129	0.00117	9.61096	2872.61
11	0.5936	0.0936	0.0400	0.01839	0.00117	15.65236	2891.88
12	0.6436	0.1436	0.0500	0.02602	0.00117	22.14809	2911.64
13	0.7136	0.2136	0.0700	0.03491	0.00117	29.71285	2923.57
14	0.8036	0.3036	0.0900	0.04406	0.00117	37.50246	2936.87
15	0.9036	0.4036	0.1000	0.05209	0.00117	44.33766	2943.62
16	1.0036	0.5036	0.1000	0.05852	0.00117	49.81073	2950.18

K	29.87 M^{-1}
Error	0.83113275 M^{-1}

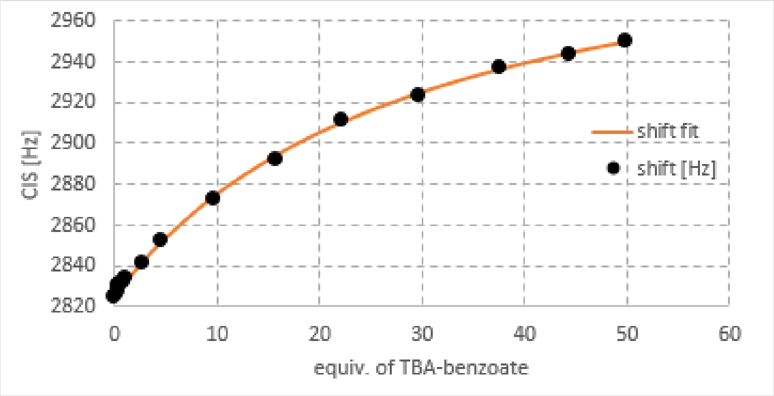

Figure S43:^{1}H NMR titration of compound **16** with TBA benzoate ($CDCl_3$, 400 MHz).

Calixarene **16** was dissolved in a specified amount of $CDCl_3$. 0.5 ml of calixarene solution was put in an NMR tube. To the calixarene solution (0.6 ml), a specific amount of tetrabutylammonium acetate (TBA-acetate) was added. The aliquots of TBA-acetate were gradually added to the NMR tube to achieve different calixarene/guest ratios (1:0.000-48.590), ensuring constant host concentration during the experiment. The complexation constant was determined by analyzing CIS of host protons using nonlinear curve-fitting procedure (program BindFit).

M(TBA-acetate)	242.47	g/mol	M(calix)	768.918	g/mol
m(TBA-acetate)	0.01655	g	m(calix)	0.00271	g
c(TBA-acetate)	0.11376	mol/l	c(calix)	0.00117	mol/l
V(CDCl3_TBA-acetat	0.6	ml	V(CDCl3_calix)	3	ml

	V (total) [m	V(addition, total) [r	V(addition) [ml]	c(TBA-acetate) [r	c(calix) [mol/l]	c(calix)/c(TBA	shift [Hz]
1	0.5000	0.0000	0.0000	0.00000	0.00117	0.00000	2825.63
2	0.5006	0.0006	0.0006	0.00014	0.00117	0.11606	2826.16
3	0.5012	0.0012	0.0006	0.00027	0.00117	0.23184	2826.33
4	0.5020	0.0020	0.0008	0.00045	0.00117	0.38579	2830.64
5	0.5028	0.0028	0.0008	0.00063	0.00117	0.53924	2830.84
6	0.5042	0.0042	0.0014	0.00095	0.00117	0.80662	2831.53
7	0.5056	0.0056	0.0014	0.00126	0.00117	1.07251	2832.60
8	0.5136	0.0136	0.0080	0.00301	0.00117	2.56410	2839.55
9	0.5236	0.0236	0.0100	0.00513	0.00117	4.36449	2848.55
10	0.5536	0.0536	0.0300	0.01101	0.00117	9.37539	2869.77
11	0.5936	0.0936	0.0400	0.01794	0.00117	15.26872	2889.34
12	0.6436	0.1436	0.0500	0.02538	0.00117	21.60524	2906.06
13	0.7136	0.2136	0.0700	0.03405	0.00117	28.98459	2922.20
14	0.8036	0.3036	0.0900	0.04298	0.00117	36.58327	2934.82
15	0.9036	0.4036	0.1000	0.05081	0.00117	43.25095	2943.82
16	1.0036	0.5036	0.1000	0.05708	0.00117	48.58988	2950.18

K	23.64	M^{-1}
Error	0.408381	M^{-1}

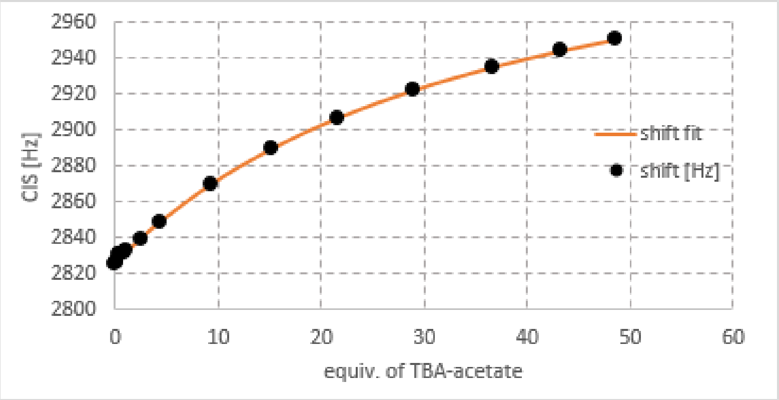

Figure S44:^{1}H NMR titration of compound **16** with TBA acetate ($CDCl_3$, 400 MHz).

5. VT ^{1}H NMR experiments

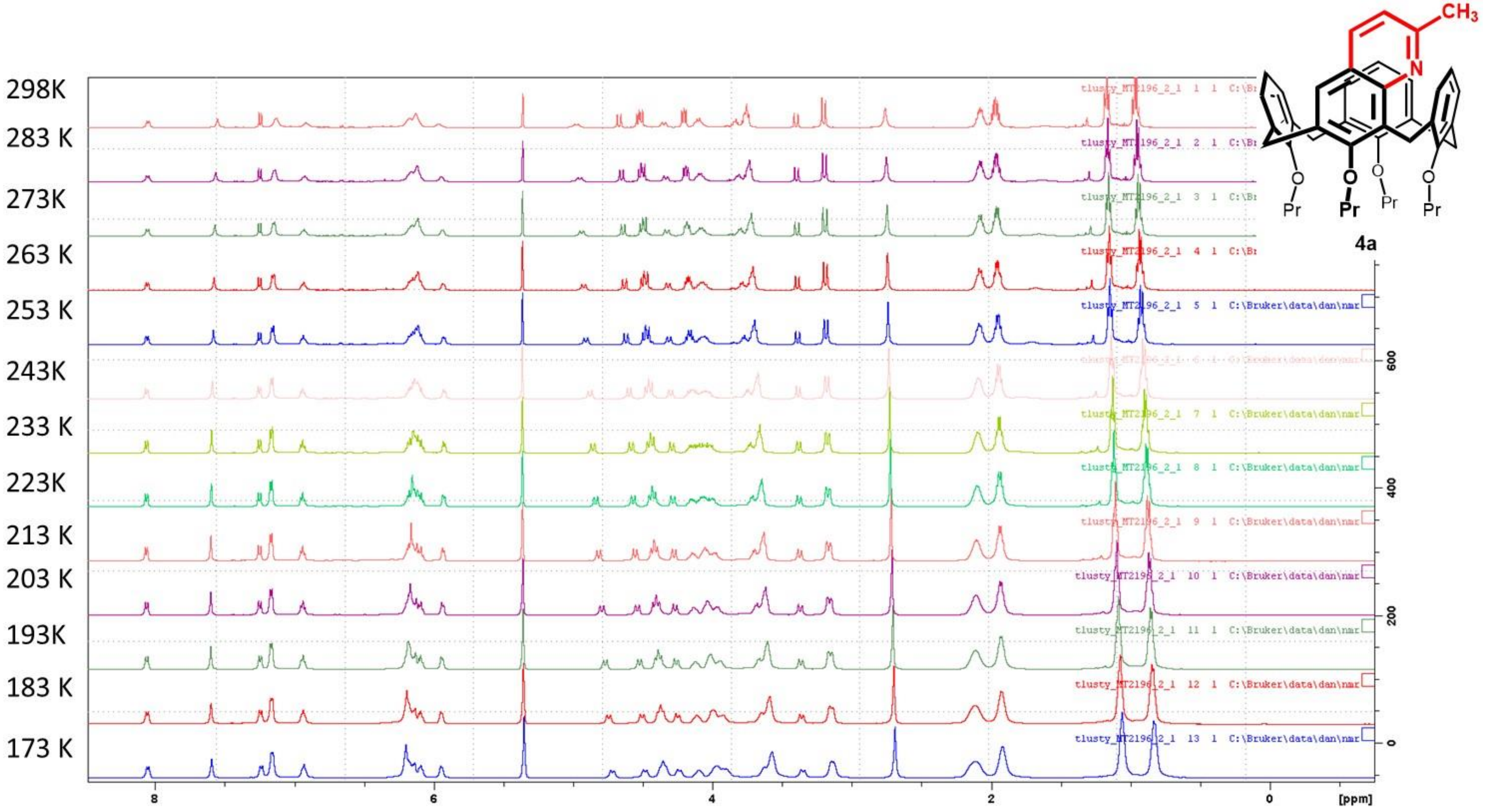

Figure S45: Variable temperature ^{1}H NMR spectra of compound **4a** (CD_2Cl_2, 500 MHz).

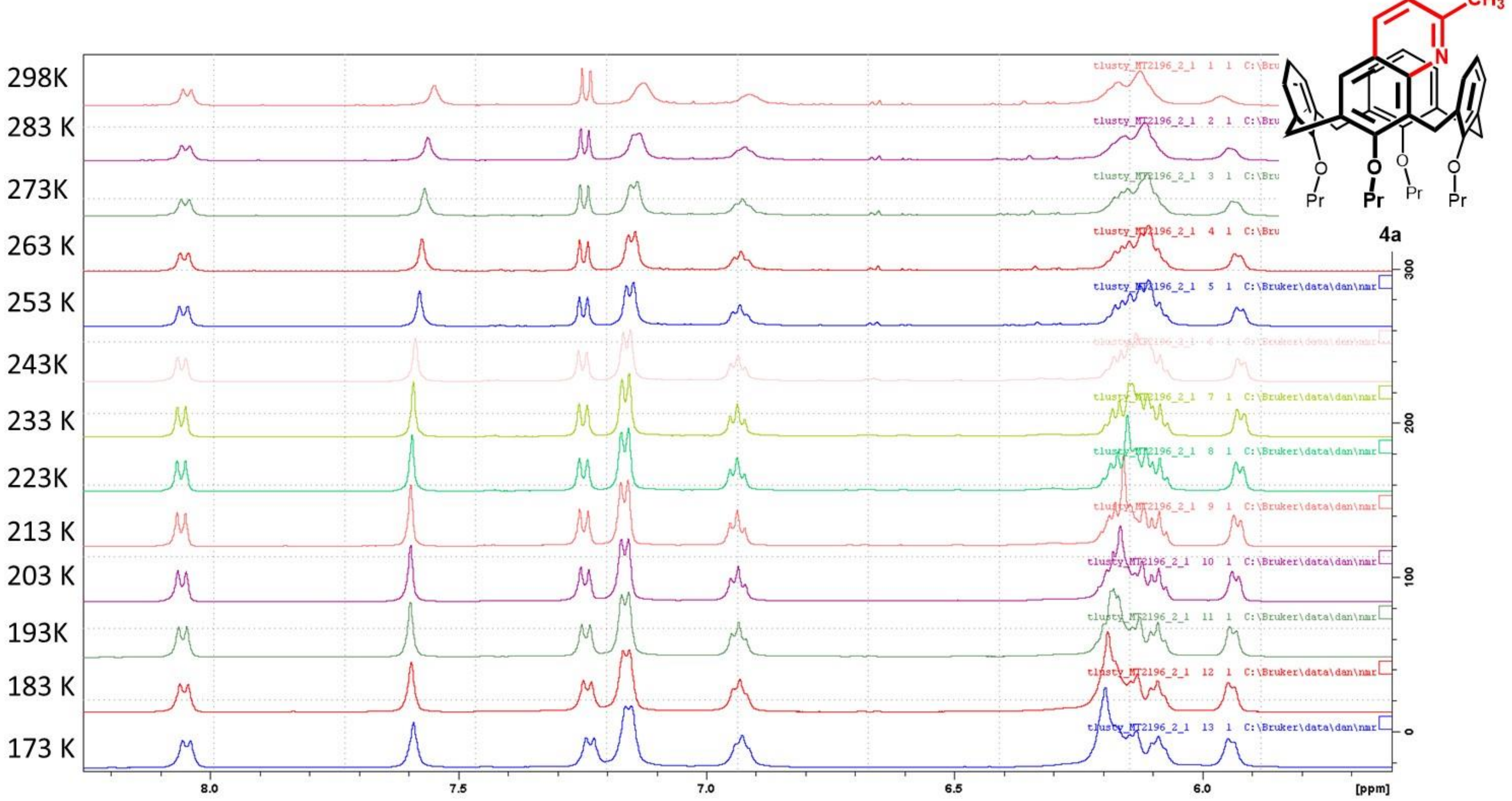

Figure S46: Variable temperature ^{1}H NMR spectra of compound **4a** (CD_2Cl_2, 500 MHz) – aromatic signals region.

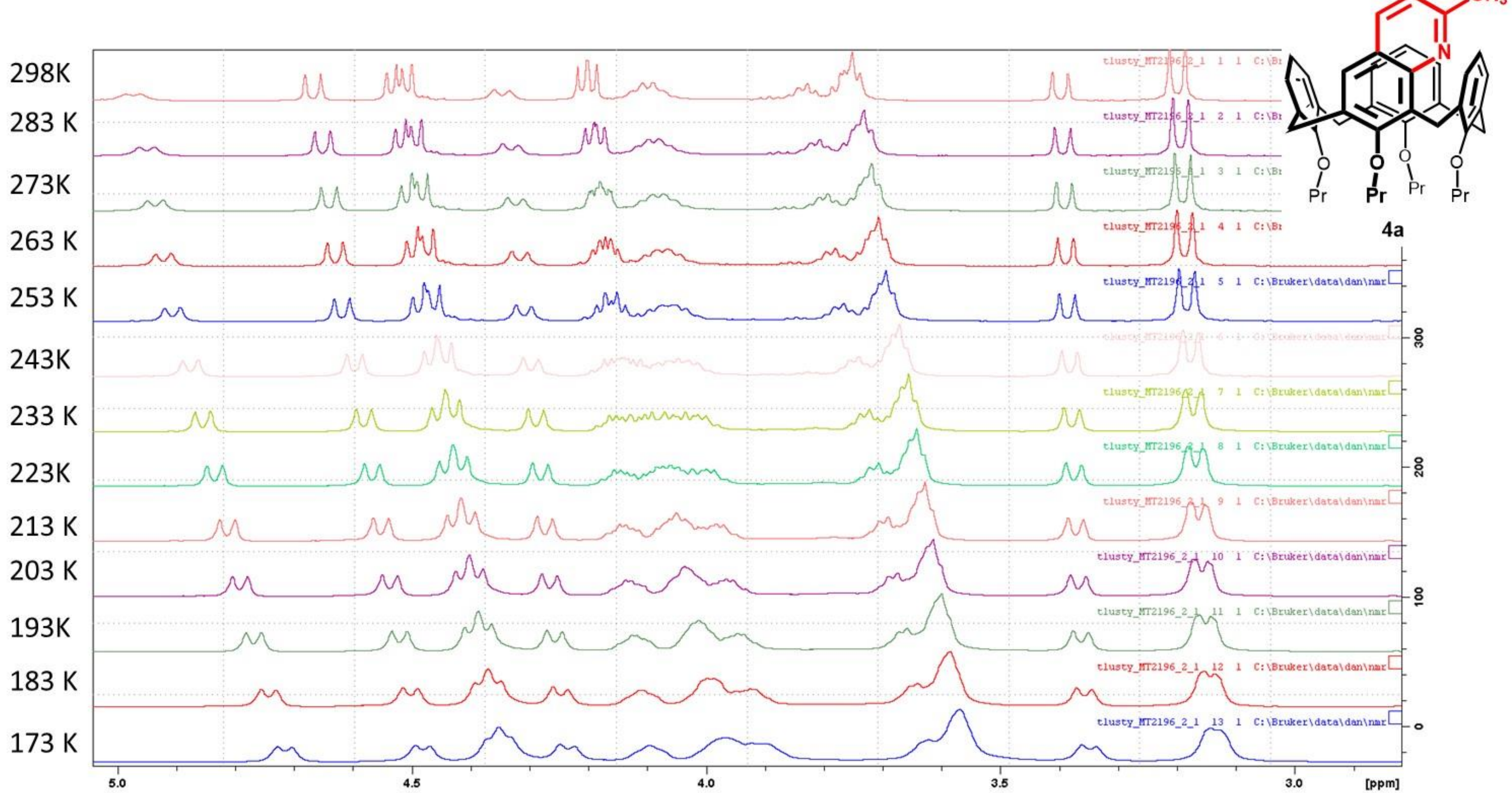

Figure S47: Variable temperature ^{1}H NMR spectra of compound **4a** (CD_2Cl_2, 500 MHz) – CH_2 signals region.

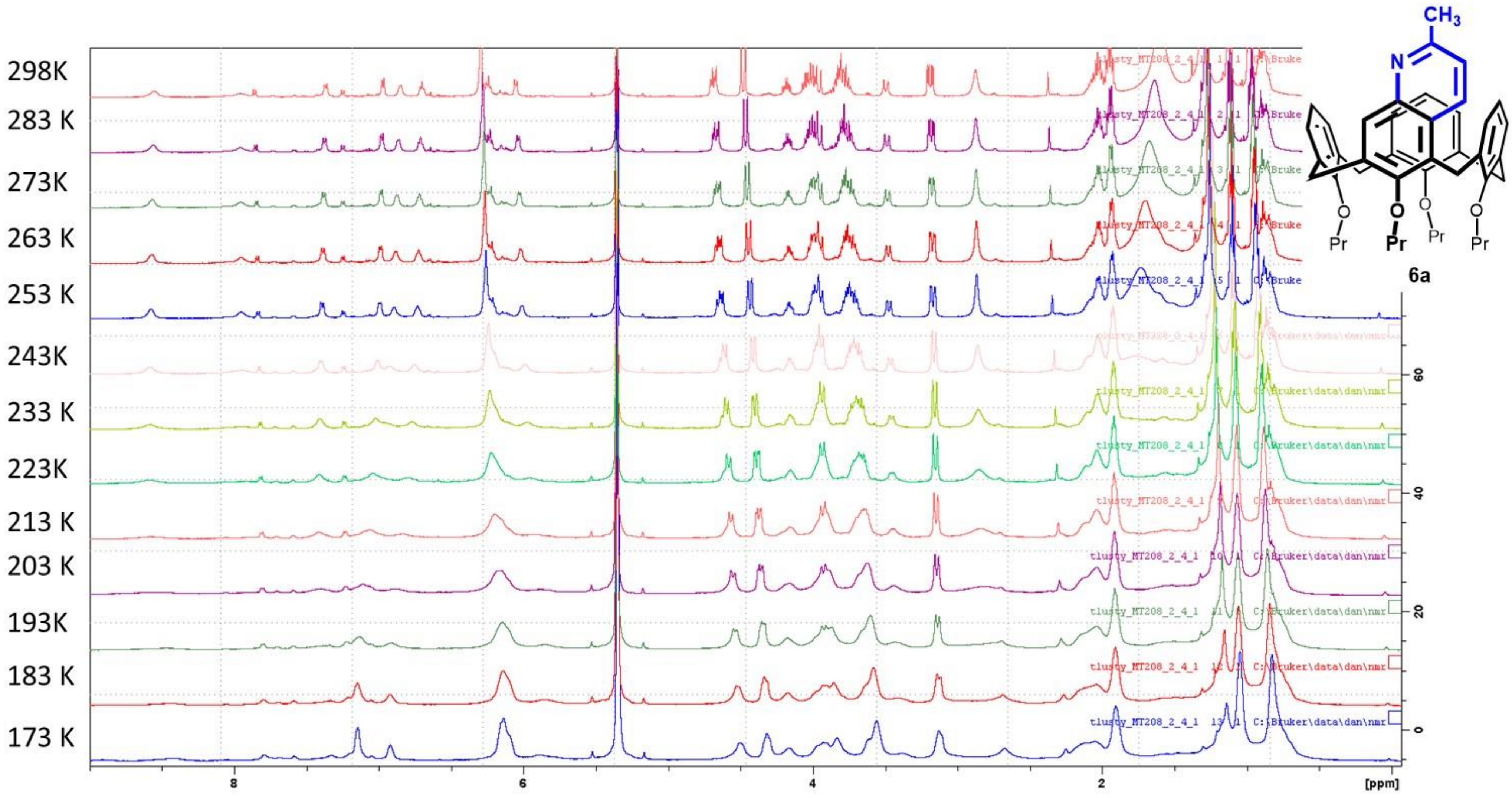

Figure S48: Variable temperature ^{1}H NMR spectra of compound **6a** (CD_2Cl_2, 500 MHz).

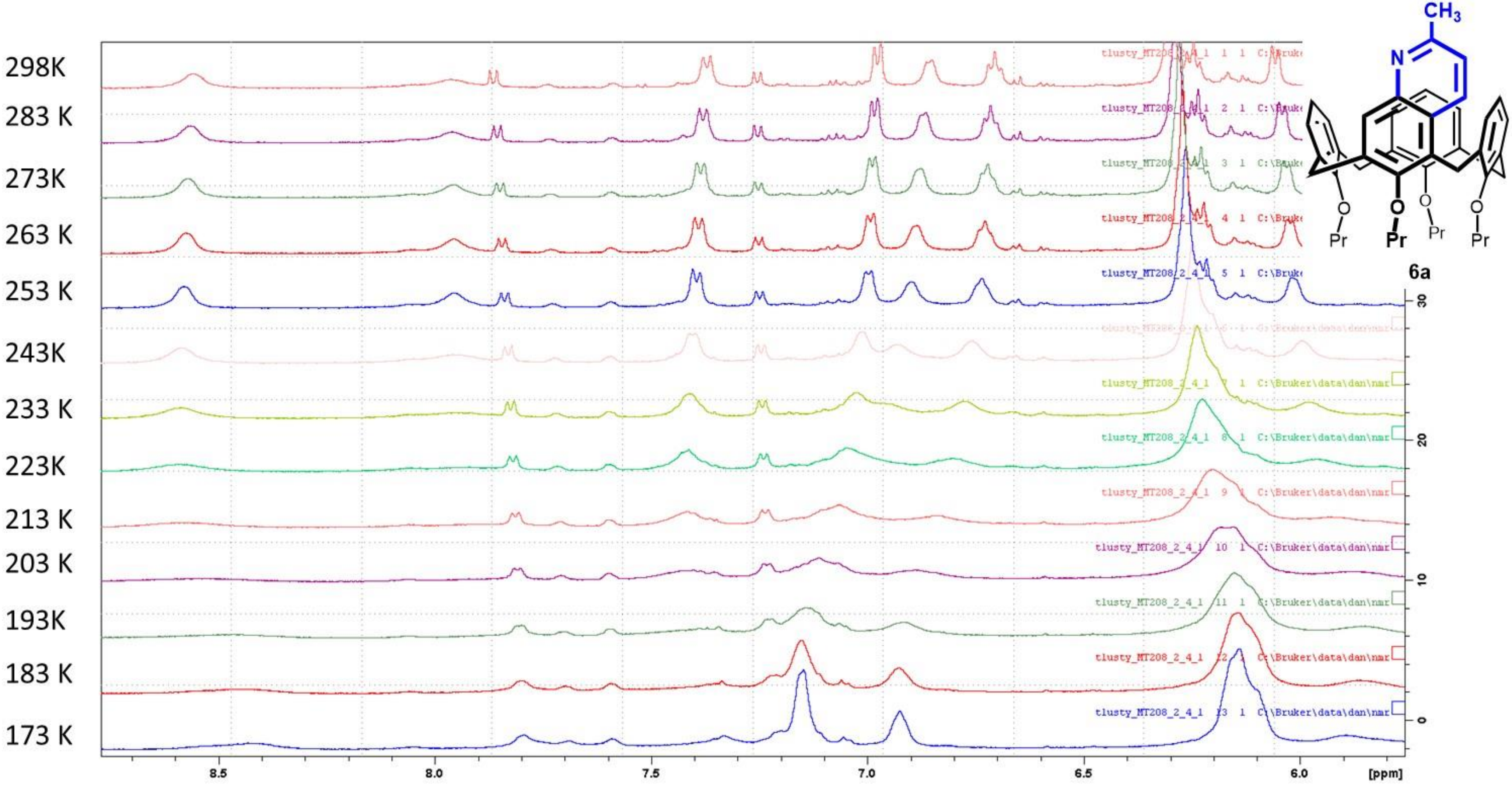

Figure S49: Variable temperature ^{1}H NMR spectra of compound **6a** (CD_2Cl_2, 500 MHz) – aromatic signals region.

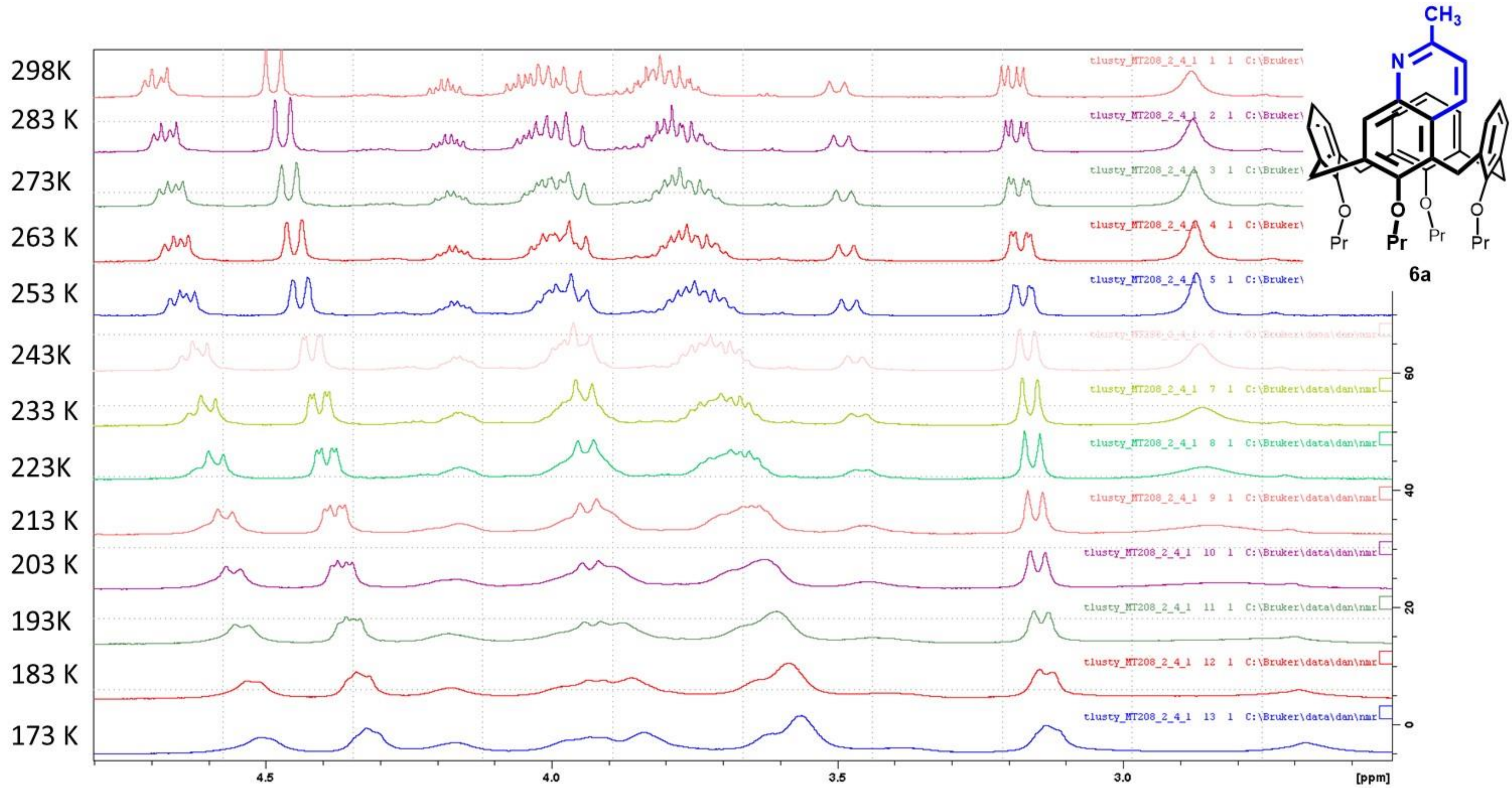

Figure S50: Variable temperature ^{1}H NMR spectra of compound **6a** (CD_2Cl_2, 500 MHz) – CH_2 signals region.
